# Applying an Empirical Hydropathic Forcefield in Refinement May Improve Low-Resolution Protein X-Ray Crystal Structures

**DOI:** 10.1371/journal.pone.0015920

**Published:** 2011-01-05

**Authors:** Vishal N. Koparde, J. Neel Scarsdale, Glen E. Kellogg

**Affiliations:** 1 Institute of Structural Biology and Drug Discovery, Virginia Commonwealth University, Richmond, Virginia, United States of America; 2 Department of Medicinal Chemistry, Virginia Commonwealth University, Richmond, Virginia, United States of America; 3 Center for the Study of Biological Complexity, Virginia Commonwealth University, Richmond, Virginia, United States of America; John Innes Centre, United Kingdom

## Abstract

**Background:**

The quality of X-ray crystallographic models for biomacromolecules refined from data obtained at high-resolution is assured by the data itself. However, at low-resolution, >3.0 Å, additional information is supplied by a forcefield coupled with an associated refinement protocol. These resulting structures are often of lower quality and thus unsuitable for downstream activities like structure-based drug discovery.

**Methodology:**

An X-ray crystallography refinement protocol that enhances standard methodology by incorporating energy terms from the HINT (Hydropathic INTeractions) empirical forcefield is described. This protocol was tested by refining synthetic low-resolution structural data derived from 25 diverse high-resolution structures, and referencing the resulting models to these structures. The models were also evaluated with global structural quality metrics, e.g., Ramachandran score and MolProbity clashscore. Three additional structures, for which only low-resolution data are available, were also re-refined with this methodology.

**Results:**

The enhanced refinement protocol is most beneficial for reflection data at resolutions of 3.0 Å or worse. At the low-resolution limit, ≥4.0 Å, the new protocol generated models with Cα positions that have RMSDs that are 0.18 Å more similar to the reference high-resolution structure, Ramachandran scores improved by 13%, and clashscores improved by 51%, all in comparison to models generated with the standard refinement protocol. The hydropathic forcefield terms are at least as effective as Coulombic electrostatic terms in maintaining polar interaction networks, and significantly more effective in maintaining hydrophobic networks, as synthetic resolution is decremented. Even at resolutions ≥4.0 Å, these latter networks are generally native-like, as measured with a hydropathic interactions scoring tool.

## Introduction

The importance of structure in understanding biomacromolecular function is well established. Applications of these structures span many disciplines, but a marquee use has been, and will likely continue to be, in the discovery of new therapeutic agents for treatment of human disease. Unfortunately, many biomacromolecules, including some of the most therapeutically relevant targets (e.g., membrane-bound proteins like G-protein coupled receptors, ion channels and efflux pumps), are not amenable to X-ray crystallography, primarily due to the difficulty of obtaining diffraction-quality crystals. NMR, the only other experimental technique that can yield near-atomic resolution models for biomacromolecules, has a different set of experimental limitations [1], [2] that are particularly evident for single proteins with molecular masses greater than 25–30 kD. Some “diffraction-quality” crystals, especially for high molecular weight or multi-protein complexes, do not diffract to sufficient resolution to produce effective target models for rational drug discovery [3]. In fact, about 25% of the protein crystal structures deposited in the RCSB protein data bank (PDB) [4], some of modest size, have resolutions of 2.5 Å or worse and the number of such structures has been increasing rapidly since 1993 [5].

As crystallographic resolution decreases, the parameter-to-observable ratio increases, i.e., the atomic coordinates and other structural model parameters are being fit to fewer experimental data, which then decreases statistical confidence in the accuracy of the refined atomic protein model [6]. Protein structural models based on low-resolution electron density maps may thus lack accuracy, and their proximity to the ”true” protein structure present in the crystal is more uncertain. Ultimately, using atomic protein models refined from low-resolution X-ray data as starting points for further studies such as drug discovery and design may well prove to be problematical or even pointless.

Recently, we coined a term – *isocrystallographic* – to describe the ensemble of alternate protonation state models for a protein or protein-ligand complex that fits the experimental structural data [7]. This ensemble was independent of resolution unless the structure was collected at high enough resolution to confidently locate all protons – at which point there would only be *one* valid structure. Here, we propose to expand the definition of an *isocrystallographic* ensemble to include all structural models consistent with the experimental electron density envelope. This ensemble is resolution-dependent since a large set of structural models will likely be consistent with low-resolution electron density envelopes, compared to a much smaller set of models at higher resolution. All of these models will likely exhibit similar refinement metrics, and it could be exceedingly difficult to choose the most biologically relevant structural model from the *isocrystallographic* ensemble. The availability of methodologies that assist in this selection of relevant atomistic protein structural models from low-resolution X-ray data will lead to an enhanced understanding of biological structure and function.

Recently, Schröder, Levitt and Brunger reported that the quality of low-resolution structural models was improved by refining against a potential function that incorporated an energy term based on deformable elastic networks [8]. Using specific distance restraints from a reference structural model to supplement standard stereochemical information (bond lengths, angles and atomic van der Waals radii) resulted in refined structural models for low-resolution data that better fit experimental structure factor amplitudes as indicated by lower R_free_ [9] values, and that also had more residues in favored regions of Ramachandran plots [10]. While the reference is ideally a high-resolution experimental structure model of a closely homologous protein, some success was also reported with modeled or predicted structures(8). This is potentially a very powerful tool for refining structural models against low-resolution X-ray data; however, its applicability may be somewhat limited since homologous reference structure models may not always be available and, with moderate-to-weak homology, selecting the most appropriate homolog and constructing optimal alignments are both formidable problems. These caveats suggest that the Schröder, Levitt and Brunger approach provides an valuable tool for a subset of proteins, but may not provide a universal solution for improving the quality of low-resolution structural models.

Here, we present a very different approach that also achieves the goal of improving structure quality; in this case by incorporating terms responsive to hydropathic interactions into the X-ray refinement target function using the empirical HINT (Hydropathic INTeractions) forcefield [11]. Our protocol does not require the existence of a previously determined high-resolution structure of a homolog, and thus is applicable to any structure. It is important to note here the differences between the HINT forcefield and conventional Newtonian molecular mechanics forcefields as used in structure optimization and dynamics annealing. While all non-covalent interactions are represented by either the Coulombic or van der Waals terms in conventional forcefields, HINT summarizes hydrophobic and polar non-covalent interactions in terms of atom-based thermodynamic parameters derived from experimental LogP_o/w_ (1-octanol/water partition coefficient) data from small molecules [11]. Partition coefficients are free energies [12] and thus HINT inherently and implicitly encodes both enthalpy and entropy in its scoring scheme. These atomistic parameters are correlated so as to calculate interaction scores that have been shown to track with free energies of association for numerous biomacromolecular systems [13], [14], [15], [16].

In the HINT model each atom-atom interaction is categorized as one of the following: (Lewis) acid-base (or the special case of hydrogen bonding) – scored favorably; acid-acid or base-base – both scored unfavorably; hydrophobic-hydrophobic – scored favorably; or hydrophobic-polar – scored unfavorably. The latter represents desolvation energy. There are interesting differences between this paradigm and Coulombic-like terms used in molecular mechanics forcefields. Hydrophobic atoms (or united atoms like –CH_3_) usually have positive, albeit small-valued, charges. This would suggest that, absent van der Waals, hydrophobic-hydrophobic interactions are unfavorable according to molecular mechanics. Similarly, since some polar atoms (generally those that are Lewis bases) have partial negative charges, their interactions with hydrophobic groups are regarded by molecular mechanics as favorable, while others (involving Lewis acids) are unfavorable. We will show here that high-resolution X-ray crystallographic structures generally support the HINT view of these interactions.

Among the various X-ray structure refinement tools available to crystallographers, CNS (Crystallography and NMR System) [17] is among the most widely used. About 30% of the X-ray structures deposited in the PDB were refined using CNS, with the large majority reporting the use of versions 1.0 or 1.1. This number is significant, as roughly an equal number of PDB entries do not report the software used for refinement and CNS has only been available since 1998. The popularity of CNS 1.1 combined with its open architecture prompted us to augment it with the HINT forcefield in order to develop a modified CNS that incorporates hydropathy in refinement. Although CNS supports the optional modeling of electrostatic interactions, the core Engh and Huber forcefield [18] does not explicitly include hydrogen bonding or electrostatic interactions in crystallographic refinement. Typically, all nonbonded interactions are modeled with a simple quadratic repulsive term in CNS, which does not compromise structural models refined against high-resolution X-ray data where atomic positions are well defined solely by experimental data. However, it likely does compromise structural models refined against low-resolution structural data where atomic positions are less well defined.

In this contribution, we show that these shortcomings can be amieliorated by including the HINT forcefield energy term in the CNS target function. To test this refinement protocol we designed a novel, rigorous test regimen to validate our approach, and, in fact, the details of our regimen are themselves a significant contribution. We demonstrate the quality of our refined structural models by validation with several commonly-used structural analysis tools.

## Results and Discussion

**Figure 1:**
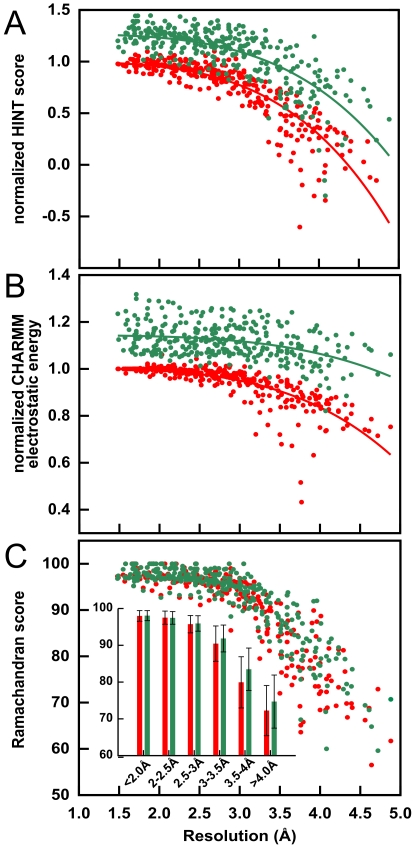
Degradation of structure quality as a function of simulated resolution: 25 high-resolution X-ray structure models refined with CNS, without (red) and with (green) electrostatics. (A) Intramolecular HINT score, normalized to that of deposited high-resolution crystal model, for CNS and CNS+electrostatics refined models. (B) Electrostatic component of CHARMM energy, normalized to that of crystal model, for CNS and CNS+electrostatics refined models. (C) Ramachandran score (percent residues in favored regions) for CNS and CNS+electrostatics refined models. The inset illustrates these same data binned by resolution ranges.

It is obvious and unassailable that current protocols for model-building and refinement based on low-resolution X-ray reflection data produce structural models of poorer quality than those based on high-resolution data. We are testing the hypothesis that these deficiencies can, at least in part, be related to the lack of well-developed hydropathic interaction networks in these models. We have sought to illustrate this point with available crystallographic data, but there is a paucity of directly comparable and unbiased structural data for proteins solved at varying resolutions. Another approach, used in this work, is to synthesize low-resolution data by truncating high-resolution data (*vide infra*) and evaluate structures refined against these data [8]. In Figure 1A (red circles) we present normalized (relative to the crystallographic structure model) intramolecular HINT scores, calculated for 309 structural models for 25 proteins refined against data truncated at resolutions between 1.48 and 4.88 Å. This score is calculated as the sum of all non-covalent intramolecular atom-atom interactions using the paradigm described above, i.e., higher scores represent *in toto* more favorable high-quality interactions within the structure. Clearly, there is a trend of an accelerating decrease in HINT score, especially for resolutions worse than 3.0 – 3.5 Å, indicating that, just as we hypothesized, these models indeed have poorer quality hydropathic interaction networks. Another evaluation of structure as a function of resolution can be obtained by calculating non-covalent energies of structure models with a molecular mechanics forcefield. The CHARMM [19] electrostatic term (Figure 1B, red circles) shows a similar trend: between 3.0 and 4.8 Å there is a more than 30% decrease in favorable electrostatic energies, relative to those in the crystallographic models, again in accord with our hypothesis. This theme is repeated with other knowledge-based structural metrics including Ramachandran scores (percentage of residues in the favored regions), as illustrated (red circles) in Figure 1C. All of these data confirm that there is a clear tendency towards decreasing structural quality as the experimental resolution of the data is decreased.

One approach to probe, and perhaps ameliorate, the disparity between structural models refined with high- and low-resolution data, is to include electrostatic interactions in X-ray refinement protocols. If electrostatics substantively improves structural quality, we can assert that compromises to polar interaction networks, e.g., hydrogen bonds or weaker, longer-range acid-base interactions, are the dominant source of structural errors in low-resolution structural models. On the other hand, partial or negligible changes in structure quality would strongly suggest that other factors are at play. In Figures 1A, 1B and 1C (green circles), we present normalized intramolecular HINT scores, normalized electrostatic energies from CHARMM and Ramachandran scores, respectively, for structures refined with the optional electrostatics protocol in CNS, which we are terming “CNS+electrostatics”. While the HINT scores (Figure 1A) are higher overall by about 25% after refinement with this protocol, the trend of decreasing HINT score with resolution is essentially unchanged. Electrostatic energy (Figure 1B) is likewise stabilized by about 15%, but even this, which essentially reports the same property used in its optimization, trends to lower values (higher energies) with lower resolution. Finally, Ramachandran scores (Figure 1C) suggest that refinement with electrostatics only modestly improves structural quality (4% improvement at 3.5–4.0 Å and 2% at ≥4.0 Å) for models from low-resolution data. The lack of significant improvement of the latter is especially notable as it is an independent and universally accepted structural metric. Furthermore, the higher overall HINT scores and lower electrostatic energies, which were both referenced to their deposited high-resolution structural models, suggests that the inclusion of electrostatics in refinement may result in models with non-native (and potentially overweighted) polar interaction networks.

In the remainder of this paper we describe the implementation and testing of a structure refinement protocol enhanced with the HINT hydropathic forcefield. It is our view that, because Coulombic electrostatic terms focus exclusively on polar components of interaction networks, refinement with electrostatics is, at best, inadequate for improving the quality of low-resolution structure models. It is important to also include terms that improve the independent and complementary hydrophobic component of the networks.

### Implementing the HINT forcefield in CNS

A modified CNS energy function was implemented: *E_total_ = E_geom_ + w_a_ E_X-ray_ + w_HINT_ E_HINT_*, where *E_geom_* accounts for the covalent, dispersion, and electrostatic energies (when activated, as in the “CNS+electrostatics” protocol) of the biomolecule, i.e., from an adaptation of the Engh and Huber forcefield [18], *E_X-ray_* represents the energy contributions from the experimental X-ray amplitudes (with relative weight *w_a_*) and *E_HINT_* is the HINT energy term (with relative weight w_HINT_). *E_HINT_* is calculated by applying a standard constant (1 kcal mol^−1^  = 515 score units [14], [20]) to the HINT score, which is the double sum over all atom pairs for two terms, ∑∑ *[a_i_ S_i_ a_j_ S_j_ exp(-r_ij_) T_ij_+50 F(r_ij_)]*, where a is the hydropathic atom constant and S is the solvent accessible surface area of atoms (*i* and *j*), *r_ij_* is the distance between these atoms, *T_ij_* is a discriminant function for polar-polar interactions, and *F* is the Levitt [21] implementation of the Lennard-Jones potential. While *w_a_* is optimized internally by CNS, the *w_HINT_* term was optimized (*vide infra*) for each refinement by identifying the value producing the lowest *R_free_*. This protocol will be referred to as “CNS+HINT” throughout this report.

### Simulation of low-resolution data sets

Assessing a protocol that purports to improve structural quality for low-resolution models requires known experimental structures of accepted high quality as references. Because there are few, if any, authentically low-resolution data sets for which high-resolution structural data also exists in the same crystal form, our approach was validated *within* the same data set. We chose 25 high-resolution (≤1.5 Å) and diverse (≤30% homology between any pair) structures from the PDB for which structure factor data was available as our reference sets. Artificial but realistic low-resolution data were synthesized using a protocol adapted from Schröder, Levitt and Brunger [8]. In addition, the validation must simulate the process of refining the structural models at each resolution without introducing bias. We deemed it unacceptable to refine the reference atomic coordinates against the simulated low-resolution structure factor data as that would almost certainly bias the resulting refined structure towards the reference structure. Thus, we generated starting models for each structure by randomly perturbing the coordinates for each atom of the deposited PDB structural model.

### Assessing the fit of refined structures to experimental reflection data

**Figure 2:**
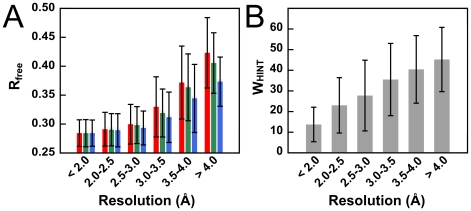
Refinement parameters of structural models: using native CNS (red), CNS+electrostatics (green) and CNS+HINT (blue). (A) Average R_free_ for structures refined within the defined simulated resolution ranges with the three protocols. The standard deviations are indicated with error bars. (B) Average w_HINT_, the weighting of the HINT term in the refinement target function, within the defined resolution ranges with indicated standard deviations.

An independent metric for assessing structural quality is provided by the fit of the calculated model structure factor amplitudes to experimental structure factor amplitudes. A protocol that aims to improve structural quality should improve the fit to experimental data, or at the very least not degrade it. In Figure 2A, we present a histogram of R_free_ values for structures refined using native CNS, CNS+electrostatics and CNS+HINT. Clearly, for the lowest resolutions, R_free_ values for structures refined using the HINT representation of non-covalent interactions are significantly lower than for structures refined with the other protocols. For higher resolution structures, the inclusion of the HINT term does not increase *R_free_*. Together, these results clearly indicate that refinement with CNS+HINT does not overfit the experimental data, and for the lowest resolution structures, improves the fit to experimental data.

Another test of the effect of the HINT term on structure is to monitor the weight assigned to the HINT term, chosen by minimizing *R_free_*, yielding the optimal structure. In Figure 2B, we present a histogram of the *w_HINT_* values for optimal structures as a function of resolution. There is a clear trend towards increasing W_HINT_ values as resolution decreases, which strongly suggests a more dominant role for the hydropathic energy term in defining atomic positions with decreasing resolution. In other words, the hydropathic term serves to restrain atomic positions in cases where atomic positions are poorly defined on the basis of experimental data alone. It should be noted that the electrostatics term in CNS+electrostatics is incorporated within *E_geom_* and its relative weight with respect to covalent terms is fixed. The contribution of electrostatics is only varied as the X-ray weighting, *w_a_*, is optimized. However, Figure 1 suggests that increasing the role of electrostatics by adaptations to the CNS energy function may be be counterproductive, while decreasing this weighting would only reduce its already minor effect.

### Assessing the model structures by superposition on the high resolution targets

**Figure 3:**
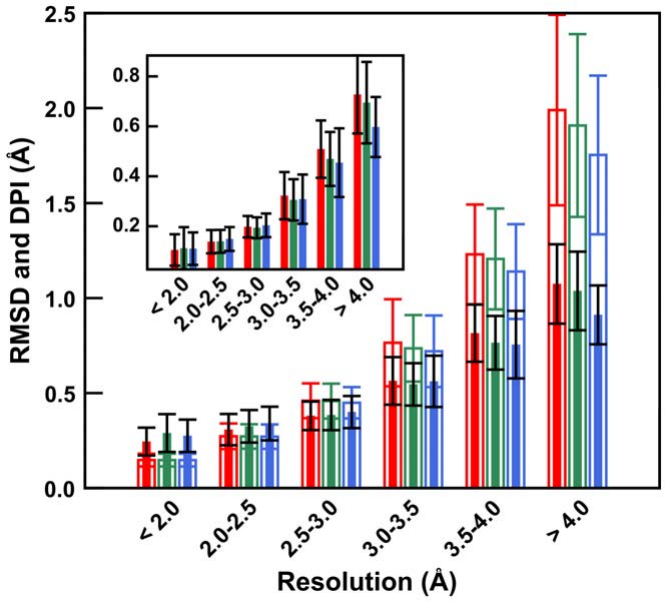
Coordinate differences between reference (deposited PDB) and re-refined models: refined using native CNS (red), CNS+electrostatics (green) and CNS+HINT (blue). Heavy atom RMSDs (solid bars, black errors) and Cruickshank Diffraction-component Precision Index (DPI, open bars, bar-colored errors) for structures refined within the defined simulated resolution ranges with the three protocols are shown. The inset provides the RMSDs for the Cα atoms only.

The “gold standard” of structural quality is probably the fit of low-resolution structural models to the experimental high-resolution reference structure. Ideally, low-resolution models should superimpose perfectly on the high-resolution target, at least for residues with well-defined electron density (i.e., those buried or involved in lattice interactions). In Figure 3, we present a histogram of heavy atom root mean squared deviations (RMSDs) for structures refined using the three protocols (inset, Cα RMSDs). It is clear that none of the protocols yields refined structures that superpose on the high-resolution target and that structural deviations increase as resolution decreases. However, for the lowest resolution structures, structures refined with CNS+HINT are, at least moderately (0.18 Å at ≥4.0 Å), closer to the high-resolution target than structures refined with the other two protocols.

Although this 15% improvement in RMSDs between structures refined with CNS+HINT and native CNS at the lowest simulated resolutions is smaller than we might have hoped, it is nonetheless significant, and there are a number of factors that may inflate the observed deviations from the high-resolution target. First, our reevaluations of refinement were automated to be performed identically. Second, surface residues (not involved in lattice contacts) have poorly resolved electron densities and are not refined well, but their atoms are included in RMSD. Third, as the assignment of waters to density can be somewhat arbitrary, we have not considered any crystallographic waters. As resolution decreases the number of waters observed decreases quite dramatically [22]. Compared to the large number (average: 235) of waters observed in the 25 reference targets, few (if any) waters would have been observed in the lower of the resolution ranges we explored. This compromises the quality of polar interaction networks for all models (regardless of protocol) since buried waters usually participate in direct or bridging hydrogen-bonding interactions and can thus affect the atomic positions of their partner atoms. Fourth, we have included explicit protons, which are required by the HINT scoring function, that were not present in any of the deposited structures. Finally, instead of the simple quadratic nonbonded term typically used in CNS crystallographic refinements, we have used the Lennard-Jones 6–12 term. Refining the deposited coordinates in the absence of hydropathic or electrostatic terms, with explicit protons and without waters, results in structures with all heavy atom RMSDs of 0.25 Å from the deposited structures, which effectively sets a floor value for RMSD comparisons. It is instructive, however, to put these RMSDs in perspective by comparison to atomic positional uncertainties, e.g., Cruickshank's Diffraction-component Precision Index (DPI) [23] values, that are also depicted in Figure 3. Clearly, RMSDs for all low-resolution cases are well within the uncertainties suggested by the DPI.

### Assessing the quality of refined structures using knowledge-based metrics

**Figure 4:**
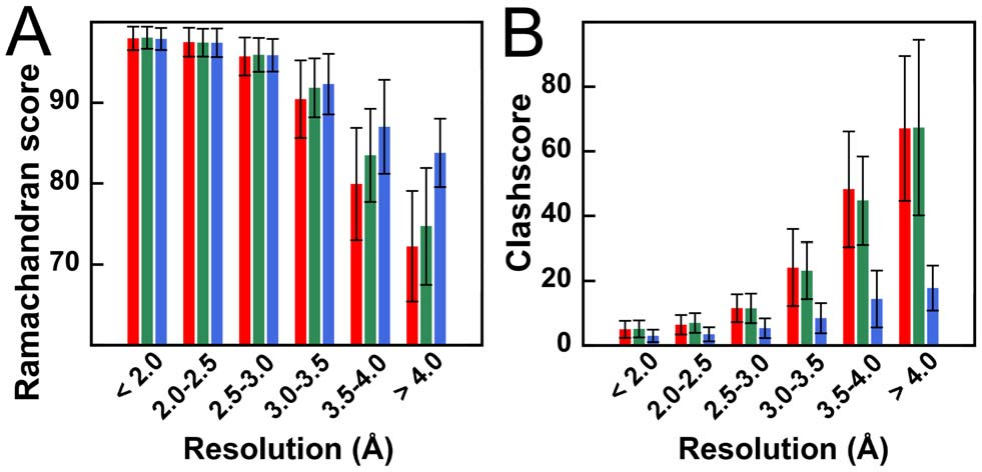
Structure quality metrics for re-refined models: using native CNS (red), CNS+electrostatics (green) and CNS+HINT (blue). (A) Ramachandran score (percent residues in favored regions) for structures refined within the defined simulated resolution ranges using the three protocols. Error bars are standard deviations. (B) Molprobity clashscores (number of steric overlaps <4 Å per 1000 atoms) within the defined resolution ranges with indicated standard deviations.

Structural quality can also be assessed by knowledge-based metrics that “rank” a structure with respect to others. Model quality, as reported by indices like the Ramachandran score or MolProbity [24] clashscore, has been shown to worsen with decreasing resolution. Histograms for Ramachandran scores (Figure 4A) and clashscores (a measure of the number of unusually short interatomic distances in a structure, Figure 4B) report the same trend: while inclusion of electrostatics alone has only a modest impact, the inclusion of the HINT representation of non-covalent interactions results in much more significant improvements in structure quality. The HINT potential, which is based on pairwise non-covalent interactions, has no “intrinsic knowledge” of preferred peptide backbone angles, yet the CNS+HINT models have its inclusion a significantly higher fraction (13% larger for resolution ≥4.0 Å) of residues in favored regions of the Ramachandran plot. Clashscores (Figure 4B) show an even more dramatic (51% at ≥4.0 Å) improvement for the CNS+HINT structures. In addition, since the clashscores for the native and CNS+electrostatics refined structures are virtually identical, the anomalously low electrostatic energies and increased HINT scores (relative to reference) for CNS+electrostatics models (Figures 1A and 1B) are, in part, an artifact of abnormally short interatomic distances between polar atoms. In contrast, the better clashscores from CNS+HINT refinement strongly suggests that this protocol results in better-defined interaction networks.

### Deconstructing the effect of the HINT term

**Figure 5:**
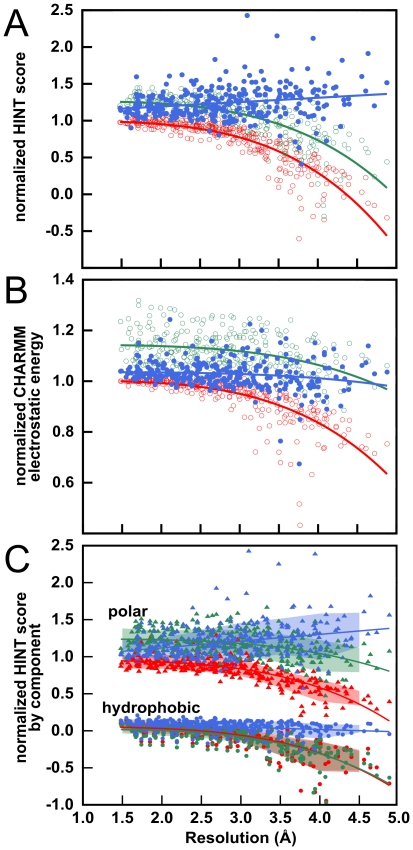
Structure quality as a function of simulated resolution: structure models refined with native CNS (red), CNS+electrostatics (green) and CNS+HINT (blue). (A) Intramolecular HINT score, normalized to that of crystal model, for models refined using the three protocols. (B) Electrostatic component of CHARMM energy, normalized to that of crystal model, for models refined using the three protocols. (C) Component decomposition of normalized intramolecular HINT score into polar and hydrophobic terms. Shaded regions indicate standard deviations of components averaged within resolution ranges.

**Figure 6:**
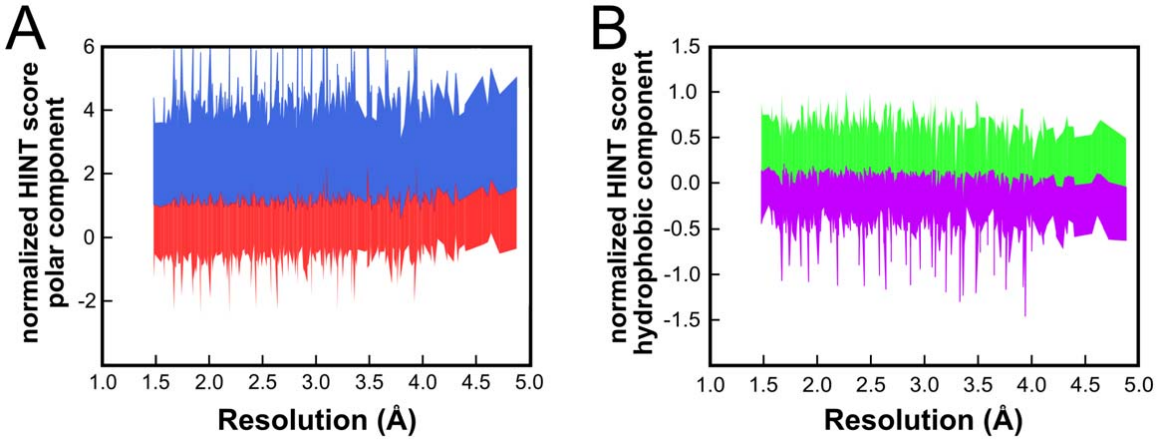
Analysis of favorable and unfavorable contributions to intramolecular HINT score: CNS+HINT refined structure models. (A) Favorable (blue, hydrogen bond and acid-base) and unfavorable (red, acid-acid and base-base) contributions to polar interaction component of HINT score. (B) Favorable (green, hydrophobic-hydrophobic) and unfavorable (violet, hydrophobic-polar) contributions to hydrophobic interaction component of HINT score.

Neither the native CNS nor the CNS+electrostatics protocols were able to maintain normalized HINT score or electrostatic energy as model resolutions decreased. Figures 5A and 5B reprise these graphs for models refined with the CNS+HINT protocol. In both cases, this protocol produces more native-like behavior (normalized value close to 1) throughout the range of resolutions. It is interesting that the relatively crude HINT “electrostatics” [11], largely based on experimental solvent partitioning of small organic molecules, perform measurably better than the CNS partial charge-based Coulombic electrostatics. This is probably because the HINT atomistic parameters are not solely electrostatic, but are scalar quantities that in principle encode all physiochemical interactions in biological media. It is revealing to deconstruct the HINT score into two components (see Figure 5C): polar, where hydrogen bonds and acid-base interactions have positive scores, while acid-acid and base-base have negative scores; and hydrophobic, where hydrophobic-hydrophobic is positive and hydrophobic-polar is negative. The HINT polar component score is similar for both the CNS+electrostatic and CNS+HINT structures, which suggests that either protocol adequately models these networks. The hydrophobic component shows remarkably consistent values with minimal scatter, but is overall, seemingly small, only 5% on average of the total score. However, this is actually the balanced sum of favorable and unfavorable terms, whose values are much larger – about +60% and −55%, respectively, of the total score (see Figure 6B). (The corresponding plot for the HINT polar term is shown in Figure 6A.) The structural integrity of these models, as evidenced above, highlights the importance of hydrophobic networks and that the HINT term effectively describes these networks.

### Understanding degradation of low-resolution structural models

**Figure 7:**
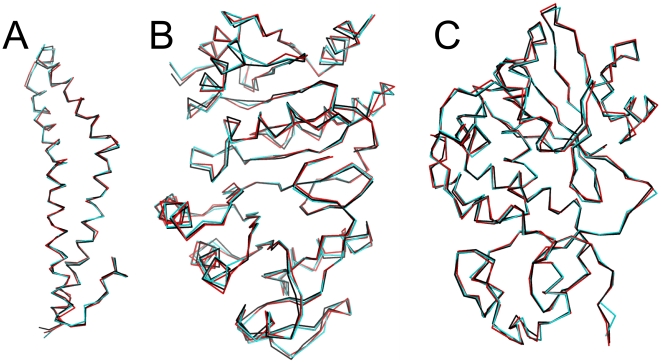
Superpositions of Cα traces: deposited structure models (black) and structure models refined with native CNS (red) and CNS+HINT (blue) at their lowest simulated resolutions. (A) 1WPA at 4.23 Å. (B) 1OI7 at 4.07 Å. (C) 1RL0 at 4.31 Å.

Although the largest deviations between low(er) resolution models and the target are generally, as expected, in solvent-exposed regions, some significant structural differences, particularly for sidechain orientations, can be found elsewhere. To further explore these differences and to better understand how interaction networks are compromised in low-resolution models, we are focusing here on three structures: 1WPA [25], 1OI7 [26] and 1RL0 [27], which are the most polar, most hydrophobic and intermediate hydrophobicity/polarity, respectively, of the 25 structures in this study. Table 1 summarizes structural and quality metrics for these structures as refined at their highest and lowest simulated resolutions. Additional data for these and the other 22 structures are available in Supporting Information (Tables S1 and S2). For all three of these structures, Cα RMSDs between structures refined with CNS+HINT and the deposited structure (see Figure 7) were between 0.5 and 0.6 Å, i.e., about 0.1 Å lower than the RMSDs for those structures refined with native CNS. For the most hydrophobic protein, 1OI7, this RMSD dropped with CNS+HINT from 0.62 to 0.50 Å.

**Table 1:** Refinement and quality statistics for re-refined high-resolution structures.

			CNS protocol		
PDB ID (resolution)	synthetic resolution (Å)	Measure	native v. 1.1	w/electrostatics	w/HINT
1WPA (1.50 Å)* [25]	1.69	*R_free_*	0.304	0.301	0.304
		*E_HINT_* (kcal mol^−1^)	−23.5	−31.2	−33.3
		Ramachandran score	100.0	100.0	100.0
		Clashscore	3.9	5.6	2.8
		Cα RMSD vs. PDB (Å)	0.09	0.11	0.12
	4.23	*R_free_*	0.552	0.468	0.464
		*E_HINT_* (kcal mol^−1^)	−7.7	−22.3	−35.8
		Ramachandran score	73.3	81.9	84.8
		Clashscore	60.6	51.1	22.8
		Cα RMSD vs. PDB (Å)	0.69	0.63	0.61
1RL0 (1.40 Å)* [27]	1.70	*R_free_*	0.286	0.284	0.289
		*E_HINT_* (kcal mol^−1^)	−65.4	−88.5	−80.6
		Ramachandran score	97.6	98.0	97.6
		Clashscore	2.7	2.7	1.2
		Cα RMSD vs. PDB (Å)	0.08	0.09	0.08
	4.31	*R_free_*	0.344	0.333	0.303
		*E_HINT_* (kcal mol^−1^)	−18.9	−56.5	−111.6
		Ramachandran score	76.7	79.4	86.2
		Clashscore	31.8	41.1	7.9
		Cα RMSD vs. PDB (Å)	0.59	0.61	0.52
1OI7 (1.23 Å)* [26]	1.49	*R_free_*	0.263	0.256	0.254
		*E_HINT_* (kcal mol^−1^)	−66.5	−77.8	−77.9
		Ramachandran score	97.0	97.4	96.6
		Clashscore	5.2	2.7	1.5
		Cα RMSD vs. PDB (Å)	0.10	0.08	0.08
	4.07	*R_free_*	0.361	0.352	0.334
		*E_HINT_* (kcal mol^−1^)	5.0	10.4	−70.0
		Ramachandran score	68.4	68.0	79.7
		Clashscore	84.2	101.9	21.7
		Cα RMSD vs. PDB (Å)	0.62	0.62	0.50

(See also Tables S1 and S2.)

*Values reported in PDB file headers.

**Figure 8:**
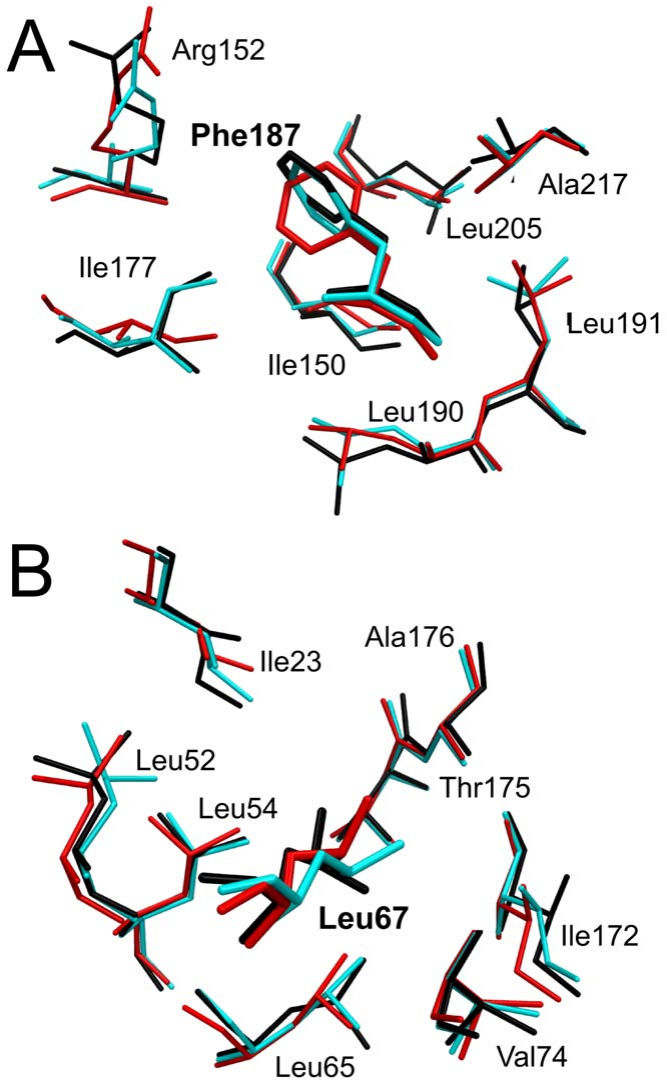
Residue sidechain superpositions: deposited structure models (black) and structure models refined with native CNS (red) and CNS+HINT (blue) at their lowest simulated resolutions. (A) Phe187 from 1OI7 at 4.07 Å. (B) Leu67 from 1RL0 at 4.31 Å.

While the backbone structures are very similar, even at low-resolution, sidechain orientations are not nearly as well-conserved. Many of the largest deviations are seen for flexible residues that are exposed to bulk solvent. However, sidechain orientations for buried hydrophobic residues in low-resolution models can also differ significantly from those in the target. Two examples are shown in Figure 8A and 8B, which are superpositions centered on residues Phe187 from 1OI7 (4.07 Å) and Leu67 from 1RL0 (4.31 Å), respectively. Inclusion of the HINT term, which explicitly encodes hydrophobic interactions, produces a Phe187 sidechain orientation that is much more similar to that observed in the deposited structure. These structural differences can be traced to differences in the underlying hydrophobic networks. This approach, however, is not a pancea that guarantees preserving the orientation of hydrophobic sidechains in low-resolution models: the orientation of the Leu67 sidechain in models refined with both native CNS and CNS+HINT differ significantly from the deposited structure. Generally, and regardless of refinement protocol, sidechain orientations are largely preserved in models at resolutions 3.0 Å and better. As resolution degrades, there is less conservation; although some, particularly non-polar, sidechain conformations are retained to lower resolutions with CNS+HINT (see Figures S1 and S2).

### Refinement of “authentic” low-resolution datasets

As a final test, we have re-refined three datasets with resolutions between 3.5 and 4.0 Å: 3GEC [28] (4.00 Å), 1ISR [29] (4.00 Å) and 1SA0 [30] (3.58 Å). The results are summarized in Table 2 where the deposited, native CNS and CNS+HINT models are compared. αβ-tubulin (1SA0), in particular, is a high-profile drug target in which we [16] and others [31], [32] have an interest, but have been held back by the rather featureless colchicine binding site ascerbated by its relatively poor crystallographic resolution. Re-refinement of the deposited tubulin-colchicine structure resulted in a model with a Ramachandran score about 5% better, and a Clashscore 17% better, than the deposited structure. The *R* and *R_free_* values are higher for our model than for that deposited, but one likely cause is that REFMAC [33], with per-domain TLS (Translation Libration and Screw) refinement [34], was used in the original refinement of this particular structure. It has been noted previously that reproducing reported *R* values for low-resolution structures can be problematical [8].

**Table 2:** Refinement and quality statistics for re-refined low-resolution structures.

PDB ID(resolut.)			CNS protocol	
	measure	PDB deposited	native v. 1.1	w/HINT†
3GEC (4.00 Å)* [28]	*R*	0.244*	0.267	0.279
	*R_free_*	0.312*	0.347	0.339
	*E_HINT_* (kcal mol^−1^)	171.1‡	−6.0	−69.6
	Ramachandran score	65.5	68.3	77.5
	clashscore	58.42	63.80	23.77
1ISR (4.00 Å)* [29]	*R*	0.237*	0.199	0.203
	*R_free_*	0.259*	0.270	0.274
	*E_HINT_* (kcal mol^−1^)	2.3‡	−51.1	−138.5
	Ramachandran score	84.8	81.8	88.1
	clashscore	104.51	73.33	72.65
1SA0 (3.58 Å)* [30]	*R*	0.232*	0.267	0.264
	*R_free_*	0.249*	0.335	0.328
	*E_HINT_* (kcal mol^−1^)	129.8‡	−161.0	−377.2
	Ramachandran score	79.8	83.6	85.1
	clashscore	18.33	2.35	1.12

*Values reported in PDB file headers.

† *w_HINT_* in all cases was 40.

‡ After correction for particularly unfavorable steric clashes.

**Figure 9:**
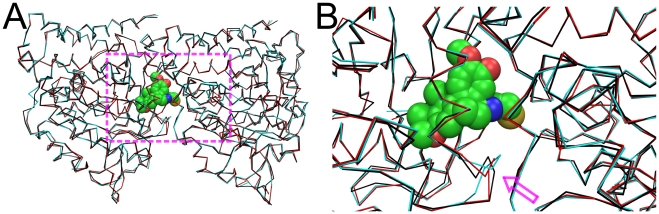
Superposition of Cα atoms in structural models for αβ-tubulin (1SA0): PDB deposited model (black), refined with native CNS (red) and CNS+HINT (blue), complexed with colchicine (spacefill, colored by atom). (A) αβ-tubulin heterodimer. Highlighted area, including colchicine ligand, is region of largest differences between the CNS+HINT and deposited structures. (B) Zoomed view of colchicine ligand binding region. Arrow indicates largest structural deviation.

Figure 9A shows a superposition of the Cα backbones for refined models of an αβ-tubulin heterodimer, while Figure 9B focuses on the region of the bound colchicine. Cα RMSDs for both the native CNS and CNS+HINT re-refined structures are ∼0.7 Å with respect to the deposited structure – similar to RMSD values (Table S1) between CNS+HINT refined low-resolution models and their high-resolution references. However, re-refinement of the tubulin structure produced some quite significant (∼2.8 Å) local deviations in Cα positions (with concomitant differences in sidechain positions) compared to the deposited structure, of which, intriguingly, the largest are localized near the colchicine binding site. We are currently exploring these new tubulin models as docking targets.

### Conclusions

We have implemented a new X-ray data refinement protocol based on CNS that relies on HINT, an empirical hydropathic forcefield, to enforce both polar and hydrophobic interaction networks for low-resolution data. Models obtained with this approach appear to have more native-like interaction networks at resolutions approaching 5 Å, as analyzed with various quality metrics, than conventionally-refined models. As currently implemented, our protocol is only applicable for protein or polynucleotide atoms in a dictionary; thus, all other atoms from ligands, water or other heterogens are refined with the default CNS protocol. Extensions to address these issues, which will likely yield even higher quality models, are currently under development. It has very recently been reported [35] that the parameterization of CNS version 1.3 yields improved low-resolution structures; we are exploring integration of our protocol to this new program.

## Methods

The analysis and refinement was performed on a dataset of 25 high-resolution X-ray crystallographic structures of proteins in the PDB (see Table S1). All 25 protein data sets satisfy the following constraints: a) X-ray resolution 1.5 Å or better; b) structure factor data are available in the PDB; c) less than 30% sequence homology with the other proteins; and d) deposited structure has no missing atoms. All water, ion and cofactor atoms were removed from the structural models for this work. CNS version 1.1 [17] was used for refinement. Its energy function was modified as described above with the HINT energy term, whose weight, *w_HINT_*, was manually optimized (to minimum *R_free_*) by performing refinement with incremental values between 10 and 100 (the trivial *w_HINT_* = 0 case is the native CNS protocol). Because HINT uses a 6–12 Lennard-Jones potential in its energy function [11], the 6–12 CNS Lennard-Jones potential was used instead of the normal quadratic potential. The HINT parameters were calculated for protein atoms using the HINT (version 3.12) dictionary method [11]; *E_HINT_* is an intramolecular energy that excludes 1–2, 1–3 and 1–4 interactions. Synthetic low-resolution datasets, ranging from ∼1.5 Å to ∼5.0 Å, were generated from high-resolution structure factor data by applying B-factor smoothing, as suggested by Schröder *et al*. [8]. Truncation was performed for each resolution at the ratio of mean intensity to the mean of its standard deviation reported at *d_min_* in the deposited structure. CCP4 [36] tools were used for file conversions and to apply the B-factor smoothing. Initial coordinates for re-refinement were generated by randomly corrupting the heavy atom positions in the deposited structures by a maximum of ±0.5 Å in each of the x, y and z-directions, before adding hydrogen atoms. Atomic scattering factors for hydrogen atoms were modified in order to eliminate any contribution to E_X-ray_. Refinement consisted of two cycles of torsion angle annealing followed by B-factor refinements. Grouped isotropic B-factor refinement was performed for resolutions worse than 2.65 Å and individual B factor refinement at higher resolutions. Further details, including refinement statistics, are given in Supporting Information (Tables S1 and S2, Figure S3).

## Supporting Information

Figure S1 **Degradation of sidechain orientation for Phe187 in 1OI7 as a function of simulated resolution**: deposited structure model (black) and structure models refined with native CNS (red) and CNS+HINT (blue). See also Figure 8A. (A) 2.84 Å. (B) 3.28 Å. (C) 3.83 Å.
(TIFF)

Figure S2 **Degradation of sidechain orientation for Leu67 in 1RL0 as a function of simulated resolution**: deposited structure model (black) and structure models refined with native CNS (red) and CNS+HINT (blue). See also Figure 8B. (A) 2.87 Å. (B) 3.18 Å. (C) 4.14 Å.
(TIFF)

Figure S3 **Flowchart summarizing data corruption and refinement protocols for native CNS, CNS+electrostatics and CNS+HINT.**
(TIFF)

Table S1 **Refinement and quality statistics for re-refined high-resolution structures.** Refinement protocols: 0 =  native CNS v. 1.1; 0+e =  CNS+electrostatics; 0+H =  CNS+HINT.
(DOC)

Table S2 **Crystallographic statistics for re-refined high-resolution structures.** Refinement protocols: 0 =  native CNS v. 1.1; 0+e =  CNS+electrostatics; 0+H =  CNS+HINT. See Table S1 for references.
(DOC)
