# Supplementary figures and images for: Applying an Empirical Hydropathic Forcefield in Refinement May Improve Low-Resolution Protein X-Ray Crystal Structures
Source: PLoS One. 2011 Jan 5;6(1):e15920. doi: 10.1371/journal.pone.0015920 (PMC3016398; doi:10.1371/journal.pone.0015920)

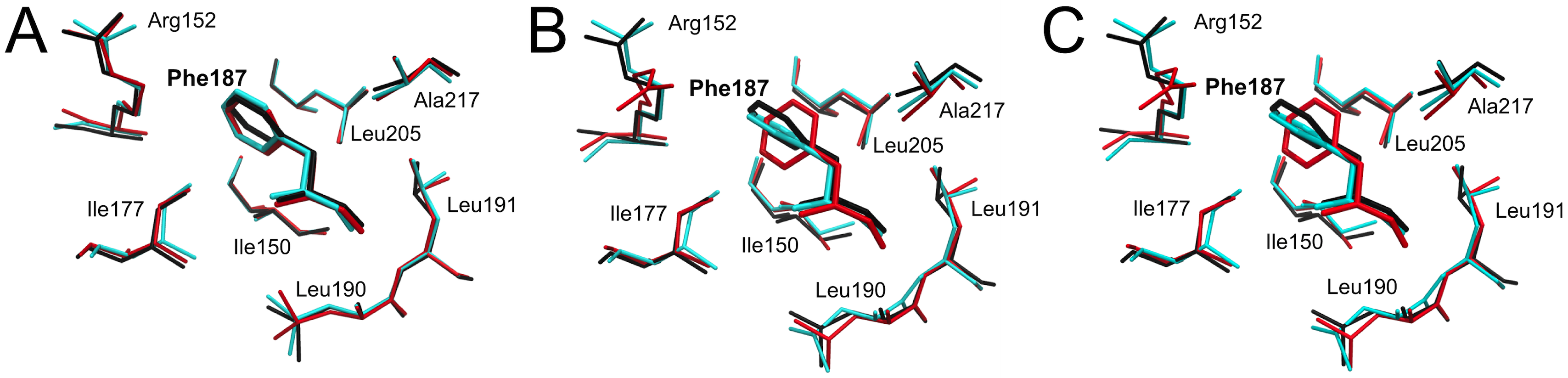

Supplement: Figure S1 — Degradation of sidechain orientation for Phe187 in 1OI7 as a function of simulated resolution: deposited structure model (black) and structure models refined with native CNS (red) and CNS+HINT (blue). See also Figure 8A. (A) 2.84 Å. (B) 3.28 Å. (C) 3.83 Å. (TIFF) [file pone.0015920.s001.tiff]

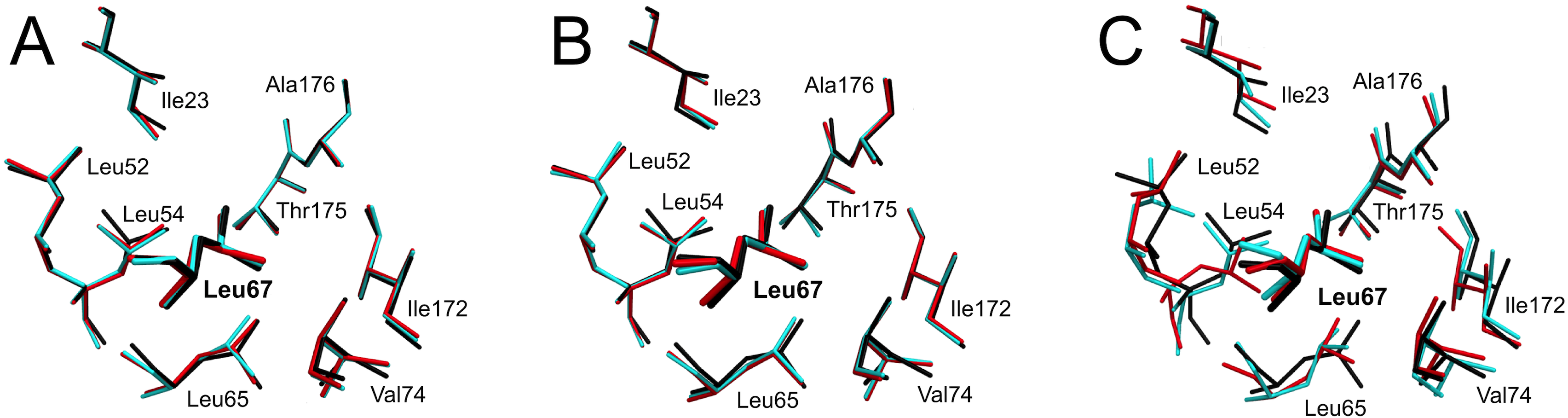

Supplement: Figure S2 — Degradation of sidechain orientation for Leu67 in 1RL0 as a function of simulated resolution: deposited structure model (black) and structure models refined with native CNS (red) and CNS+HINT (blue). See also Figure 8B. (A) 2.87 Å. (B) 3.18 Å. (C) 4.14 Å. (TIFF) [file pone.0015920.s002.tiff]

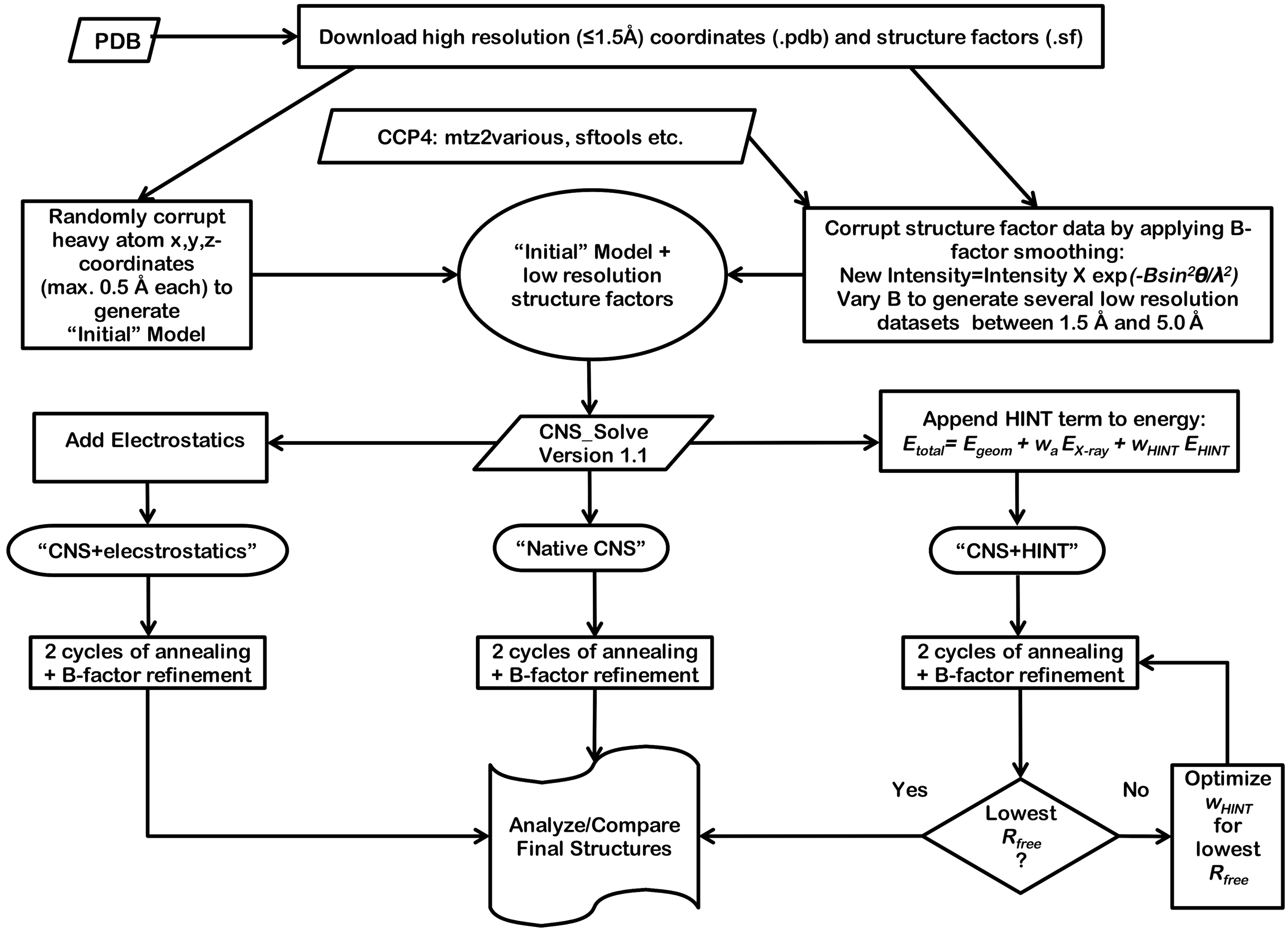

Supplement: Figure S3 — Flowchart summarizing data corruption and refinement protocols for native CNS, CNS+electrostatics and CNS+HINT. (TIFF) [file pone.0015920.s003.tiff]
