# Supplementary material for: Applying an Empirical Hydropathic Forcefield in Refinement May Improve Low-Resolution Protein X-Ray Crystal Structures
Source: PLoS One. 2011 Jan 5;6(1):e15920. doi: 10.1371/journal.pone.0015920 (PMC3016398; doi:10.1371/journal.pone.0015920)
Supplement: Table S1 — Refinement and quality statistics for re-refined high-resolution structures. Refinement protocols: 0 = native CNS v. 1.1; 0+e = CNS+electrostatics; 0+H = CNS+HINT. (DOC) [file pone.0015920.s004.doc]

PDB ID (ref)	Synth. Res. (Å)	w_{HINT}	RMSD vs. PDB (Å): Cα (all atom)			R (R_{free})			Ramachandran score			Clashscore			E_{HINT} (kcal mol^{-1})		
			0	0+e	0+H	0	0+e	0+H	0	0+e	0+H	0	0+e	0+H	0	0+e	0+H
1NA5 (S1)	1.73	10	0.10 (0.36)	0.08 (0.34)	0.09 (0.43)	0.252 (0.283)	0.254 (0.281)	0.253 (0.282)	97.4	97.4	97.4	5.1	6.4	3.2	-50.9	-72.1	-66.6
	1.89	10	0.10 (0.35)	0.09 (0.39)	0.09 (0.44)	0.249 (0.288)	0.251 (0.288)	0.249 (0.287)	97.4	97.4	97.4	5.1	6.4	3.5	-49.1	-72.2	-65.3
	2.07	30	0.10 (0.35)	0.10 (0.43)	0.12 (0.44)	0.244 (0.289)	0.246 (0.290)	0.251 (0.290)	97.4	96.9	97.4	3.5	5.5	2.9	-50.4	-69.5	-74.7
	2.26	10	0.11 (0.37)	0.12 (0.41)	0.12 (0.45)	0.237 (0.281)	0.239 (0.284)	0.238 (0.287)	97.9	97.9	97.4	5.1	5.8	3.5	-51.0	-71.7	-66.0
	2.48	10	0.15 (0.39)	0.15 (0.42)	0.14 (0.49)	0.231 (0.275)	0.232 (0.279)	0.230 (0.280)	95.9	96.4	96.9	6.8	8.4	4.5	-48.8	-68.0	-66.6
	2.65	10	0.16 (0.42)	0.17 (0.45)	0.16 (0.46)	0.223 (0.312)	0.224 (0.306)	0.225 (0.301)	95.9	96.4	94.8	8.4	9.6	5.1	-48.5	-68.9	-65.2
	3.00	20	0.20 (0.50)	0.22 (0.50)	0.21 (0.55)	0.205 (0.315)	0.205 (0.315)	0.207 (0.308)	96.9	95.9	96.9	12.2	13.8	6.8	-43.4	-67.4	-68.1
	3.16	30	0.25 (0.59)	0.26 (0.59)	0.27 (0.60)	0.219 (0.337)	0.224 (0.332)	0.224 (0.335)	92.2	92.7	95.9	23.8	28.3	8.7	-41.8	-61.1	-76.0
	3.33	20	0.29 (0.59)	0.30 (0.65)	0.29 (0.61)	0.218 (0.322)	0.217 (0.316)	0.215 (0.316)	87.6	91.7	91.7	23.5	26.7	10.9	-40.1	-60.9	-67.3
	3.72	30	0.36 (0.67)	0.36 (0.67)	0.34 (0.70)	0.195 (0.331)	0.198 (0.327)	0.200 (0.328)	84.5	89.1	91.7	39.9	37.6	8.0	-33.5	-57.7	-72.6
	4.09	30	0.58 (0.95)	0.60 (0.87)	0.49 (0.98)	0.263 (0.403)	0.254 (0.386)	0.241 (0.371)	68.9	73.6	80.8	76.8	66.5	23.5	-13.9	-41.2	-73.3
	4.40	20	0.70 (1.13)	0.76 (0.95)	0.64 (1.07)	0.326 (0.473)	0.301 (0.444)	0.280 (0.433)	68.9	77.2	78.8	78.1	68.2	27.3	-11.3	-40.7	-61.1
	4.64	50	1.00 (1.38)	1.01 (1.03)	0.65 (1.34)	0.349 (0.520)	0.345 (0.497)	0.241 (0.380)	56.5	64.8	82.9	111.2	105.4	11.2	6.1	-28.2	-95.5
1TJE (S2)	1.73	10	0.08 (0.20)	0.08 (0.20)	0.08 (0.25)	0.255 (0.281)	0.257 (0.281)	0.256 (0.282)	96.8	96.4	96.8	4.2	4.8	2.5	-53.0	-73.4	-64.0
	1.95	10	0.10 (0.19)	0.10 (0.19)	0.10 (0.22)	0.245 (0.284)	0.246 (0.285)	0.246 (0.287)	96.4	96.4	95.9	5.1	6.2	2.8	-51.4	-69.8	-62.8
	2.19	10	0.12 (0.24)	0.13 (0.24)	0.13 (0.27)	0.238 (0.276)	0.239 (0.275)	0.239 (0.277)	95.9	96.8	95.5	4.0	6.5	3.1	-51.0	-69.8	-60.7
	2.45	20	0.14 (0.27)	0.15 (0.26)	0.15 (0.27)	0.229 (0.280)	0.231 (0.277)	0.232 (0.276)	95.9	95.9	94.6	7.7	7.1	3.1	-48.0	-69.3	-65.7
	2.66	10	0.17 (0.28)	0.17 (0.27)	0.17 (0.31)	0.214 (0.289)	0.214 (0.293)	0.215 (0.295)	95.5	96.4	95.5	8.2	8.8	7.1	-48.2	-67.4	-60.7
	2.84	20	0.19 (0.31)	0.19 (0.31)	0.19 (0.33)	0.206 (0.295)	0.207 (0.291)	0.209 (0.296)	94.1	94.6	94.6	14.4	14.4	7.9	-44.0	-64.6	-64.8
	3.00	30	0.21 (0.37)	0.22 (0.37)	0.22 (0.37)	0.197 (0.296)	0.200 (0.289)	0.202 (0.288)	94.6	95.0	93.7	16.4	18.1	4.2	-45.7	-66.8	-70.7
	3.17	20	0.23 (0.38)	0.24 (0.38)	0.22 (0.39)	0.181 (0.270)	0.180 (0.278)	0.185 (0.266)	93.7	94.6	94.1	13.9	15.9	6.8	-43.9	-67.5	-64.5
	3.31	50	0.27 (0.47)	0.28 (0.48)	0.26 (0.48)	0.200 (0.316)	0.195 (0.302)	0.209 (0.292)	89.6	94.1	93.2	22.9	23.8	5.1	-40.0	-60.2	-81.9
	3.48	20	0.33 (0.61)	0.35 (0.55)	0.32 (0.58)	0.207 (0.323)	0.201 (0.295)	0.204 (0.297)	86.9	88.7	91.9	28.3	28.6	11.9	-36.4	-59.9	-66.0
	3.87	60	0.44 (0.82)	0.49 (0.74)	0.40 (0.75)	0.214 (0.376)	0.208 (0.364)	0.215 (0.344)	77.5	83.3	90.1	58.6	55.2	9.1	-20.4	-50.8	-90.0
	4.23	60	0.65 (1.01)	0.68 (0.81)	0.55 (1.03)	0.283 (0.452)	0.269 (0.406)	0.226 (0.367)	81.5	80.6	84.7	56.7	42.8	18.1	-10.3	-45.0	-70.7
	4.56	50	0.75 (1.20)	0.85 (1.00)	0.66 (1.14)	0.373 (0.496)	0.312 (0.465)	0.306 (0.421)	66.2	69.4	82.4	66.9	66.3	9.6	-1.5	-38.7	-84.4
	4.88	60	0.94 (1.49)	1.02 (1.18)	0.84 (1.39)	0.308 (0.499)	0.306 (0.504)	0.291 (0.445)	61.7	70.7	76.1	115.9	93.2	17.6	16.8	-23.3	-80.0
3CTG (S3)	1.71	10	0.09 (0.19)	0.08 (0.23)	0.09 (0.23)	0.273 (0.311)	0.275 (0.313)	0.273 (0.308)	100.0	100.0	100.0	3.0	4.7	2.4	-20.9	-25.8	-25.6
	1.93	10	0.09 (0.20)	0.09 (0.20)	0.09 (0.22)	0.269 (0.318)	0.269 (0.320)	0.268 (0.318)	100.0	100.0	100.0	2.4	3.6	1.2	-20.6	-22.7	-23.0
	2.20	10	0.11 (0.23)	0.10 (0.24)	0.11 (0.24)	0.256 (0.300)	0.261 (0.299)	0.259 (0.297)	100.0	100.0	100.0	1.8	5.3	3.6	-19.8	-22.2	-22.9
	2.46	10	0.13 (0.27)	0.13 (0.28)	0.13 (0.28)	0.254 (0.300)	0.255 (0.302)	0.255 (0.298)	100.0	100.0	100.0	7.7	8.3	0.6	-20.3	-23.5	-24.4
	2.69	10	0.15 (0.30)	0.16 (0.28)	0.16 (0.30)	0.244 (0.320)	0.242 (0.319)	0.243 (0.314)	99.1	99.1	97.2	11.3	8.9	4.7	-18.3	-21.0	-21.5
	2.93	60	0.17 (0.34)	0.17 (0.36)	0.18 (0.34)	0.235 (0.294)	0.234 (0.293)	0.244 (0.280)	96.2	95.3	96.2	11.9	12.4	3.0	-16.3	-21.1	-28.9
	3.14	10	0.23 (0.51)	0.24 (0.47)	0.24 (0.49)	0.256 (0.330)	0.256 (0.329)	0.254 (0.299)	95.3	97.2	95.3	21.3	17.8	7.7	-13.3	-18.6	-20.1
	3.38	40	0.25 (0.60)	0.32 (0.53)	0.25 (0.49)	0.250 (0.359)	0.254 (0.336)	0.259 (0.326)	90.6	91.5	97.2	21.9	24.3	8.9	-15.6	-17.6	-25.8
	3.59	60	0.38 (0.64)	0.38 (0.62)	0.35 (0.65)	0.251 (0.347)	0.254 (0.341)	0.255 (0.297)	90.6	90.6	92.5	30.8	36.8	7.1	-9.3	-12.4	-31.6
1UAI (S4)	1.48	10	0.06 (0.16)	0.05 (0.16)	0.06 (0.18)	0.238 (0.260)	0.239 (0.263)	0.240 (0.263)	97.3	96.8	97.3	8.1	6.9	4.8	-43.2	-53.1	-51.8
	1.58	10	0.07 (0.16)	0.06 (0.17)	0.06 (0.17)	0.239 (0.261)	0.240 (0.262)	0.240 (0.263)	97.3	96.8	97.3	7.8	6.9	4.8	-42.6	-52.7	-51.5
	1.85	10	0.09 (0.17)	0.08 (0.18)	0.08 (0.18)	0.236 (0.275)	0.237 (0.275)	0.237 (0.276)	96.8	97.3	97.3	7.8	9.0	6.3	-43.4	-54.6	-51.2
	2.17	10	0.12 (0.19)	0.12 (0.19)	0.12 (0.19)	0.231 (0.275)	0.231 (0.276)	0.232 (0.275)	95.9	97.3	96.4	8.4	7.5	6.3	-41.3	-52.9	-48.9
	2.52	60	0.17 (0.27)	0.15 (0.34)	0.18 (0.28)	0.219 (0.280)	0.219 (0.280)	0.244 (0.277)	95.9	95.9	95.0	11.1	11.7	5.1	-39.6	-51.5	-64.1
	2.65	60	0.18 (0.30)	0.17 (0.38)	0.19 (0.30)	0.214 (0.293)	0.215 (0.294)	0.241 (0.293)	94.1	94.1	93.7	10.8	12.0	6.0	-40.6	-50.6	-64.9
	2.78	60	0.18 (0.29)	0.17 (0.38)	0.20 (0.32)	0.202 (0.290)	0.204 (0.290)	0.233 (0.284)	92.3	93.2	92.3	13.5	13.5	3.3	-37.8	-48.0	-64.0
	2.92	10	0.21 (0.34)	0.21 (0.34)	0.20 (0.34)	0.196 (0.315)	0.195 (0.316)	0.196 (0.308)	91.0	91.4	90.0	19.7	19.4	14.1	-32.7	-47.3	-44.4
	3.06	60	0.26 (0.46)	0.26 (0.43)	0.23 (0.45)	0.191 (0.302)	0.187 (0.281)	0.211 (0.285)	91.0	91.4	94.1	23.6	25.7	4.5	-31.4	-46.2	-62.6
	3.20	60	0.29 (0.46)	0.29 (0.44)	0.25 (0.43)	0.188 (0.295)	0.188 (0.288)	0.208 (0.280)	88.2	90.0	89.6	29.0	27.5	5.1	-24.6	-40.6	-61.7
	3.35	60	0.34 (0.55)	0.36 (0.52)	0.29 (0.52)	0.189 (0.335)	0.187 (0.336)	0.200 (0.310)	86.0	86.9	87.8	33.2	29.3	10.5	-20.9	-39.4	-63.4
	3.52	10	0.40 (0.63)	0.40 (0.61)	0.38 (0.62)	0.182 (0.317)	0.184 (0.317)	0.184 (0.311)	80.1	81.0	81.0	42.2	43.1	30.2	-15.6	-31.6	-35.0

PDB ID (ref)	Synth. Res. (Å)	w_{HINT}	RMSD vs. PDB (Å): Cα (all atom)			R (R_{free})			Ramachandran score			Clashscore			E_{HINT} (kcal mol^{-1})		
			0	0+e	0+H	0	0+e	0+H	0	0+e	0+H	0	0+e	0+H	0	0+e	0+H
1UAI (cont.)	3.65	50	0.48 (0.85)	0.60 (0.64)	0.41 (0.72)	0.218 (0.404)	0.207 (0.373)	0.215 (0.339)	73.8	80.5	86.0	67.9	48.2	16.1	-1.5	-32.7	-59.9
	3.77	60	0.54 (0.91)	0.58 (0.67)	0.43 (0.80)	0.220 (0.377)	0.193 (0.343)	0.212 (0.338)	75.1	79.2	84.6	66.4	52.0	13.8	2.2	-26.4	-59.5
	3.92	60	0.50 (0.86)	0.62 (0.67)	0.41 (0.75)	0.199 (0.366)	0.190 (0.355)	0.202 (0.355)	77.4	76.0	81.4	56.5	47.9	9.6	-5.8	-31.3	-67.3
3KB5 (S5)	1.80	10	0.21 (0.39)	0.19 (0.35)	0.17 (0.39)	0.256 (0.291)	0.260 (0.294)	0.261 (0.290)	94.8	94.2	94.2	11.8	12.2	8.9	-38.6	-44.4	-44.8
	1.91	10	0.17 (0.38)	0.19 (0.42)	0.18 (0.37)	0.253 (0.294)	0.251 (0.294)	0.245 (0.289)	94.2	96.9	94.8	11.2	13.1	5.6	-38.5	-46.0	-50.6
	2.02	50	0.18 (0.42)	0.18 (0.55)	0.18 (0.40)	0.250 (0.289)	0.248 (0.288)	0.256 (0.292)	93.2	92.7	92.7	13.5	15.1	6.6	-38.1	-45.5	-58.7
	2.13	10	0.18 (0.42)	0.17 (0.45)	0.17 (0.42)	0.242 (0.280)	0.247 (0.285)	0.246 (0.282)	94.8	93.7	94.8	11.2	13.1	11.8	-34.9	-43.3	-44.1
	2.25	20	0.17 (0.40)	0.17 (0.43)	0.17 (0.41)	0.237 (0.295)	0.240 (0.295)	0.236 (0.287)	92.7	93.2	94.8	13.8	15.4	8.5	-34.8	-42.4	-48.0
	2.39	20	0.20 (0.44)	0.19 (0.46)	0.24 (0.43)	0.235 (0.286)	0.230 (0.285)	0.236 (0.283)	92.7	93.2	92.7	18.1	15.4	8.5	-32.9	-44.7	-43.5
	2.51	50	0.22 (0.45)	0.21 (0.56)	0.22 (0.44)	0.224 (0.287)	0.226 (0.282)	0.243 (0.277)	91.1	92.7	93.2	19.1	19.4	8.9	-31.9	-39.1	-58.6
	2.62	10	0.23 (0.47)	0.24 (0.47)	0.23 (0.45)	0.221 (0.300)	0.223 (0.300)	0.233 (0.300)	92.1	94.2	93.2	20.1	19.4	15.1	-32.5	-40.7	-37.4
	2.87	20	0.23 (0.53)	0.29 (0.44)	0.23 (0.44)	0.213 (0.312)	0.223 (0.306)	0.214 (0.290)	91.6	91.6	91.1	25.3	28.6	13.8	-30.5	-36.1	-42.1
	3.13	50	0.28 (0.54)	0.26 (0.58)	0.27 (0.54)	0.222 (0.309)	0.225 (0.308)	0.233 (0.295)	90.6	90.6	91.1	27.9	26.6	11.8	-25.1	-35.5	-54.1
	3.37	40	0.40 (0.77)	0.41 (0.69)	0.37 (0.73)	0.255 (0.394)	0.238 (0.358)	0.257 (0.372)	81.2	83.2	82.2	56.2	50.9	20.7	-9.6	-19.7	-48.4
	3.59	20	0.41 (0.84)	0.44 (0.89)	0.42 (0.84)	0.236 (0.375)	0.254 (0.403)	0.255 (0.395)	77.5	80.1	80.6	76.9	69.0	26.6	-12.6	-24.0	-48.1
	3.79	60	0.46 (0.85)	0.53 (0.79)	0.45 (0.84)	0.303 (0.423)	0.302 (0.425)	0.286 (0.402)	80.1	80.1	81.7	59.1	59.8	17.4	-12.2	-20.5	-57.1
	4.00	50	0.65 (1.05)	0.62 (0.94)	0.58 (1.04)	0.260 (0.392)	0.253 (0.387)	0.256 (0.377)	78.5	75.9	82.7	102.2	86.4	27.3	6.5	-6.1	-54.2
2I49 (S6)	1.57	10	0.05 (0.12)	0.06 (0.14)	0.06 (0.16)	0.243 (0.250)	0.229 (0.237)	0.242 (0.251)	96.2	97.2	96.2	5.2	3.6	4.5	-112.3	-141.9	-121.4
	1.76	10	0.06 (0.14)	0.07 (0.14)	0.07 (0.15)	0.236 (0.258)	0.225 (0.251)	0.236 (0.259)	96.7	97.2	96.7	4.7	4.4	5.0	-112.0	-143.7	-121.5
	1.92	10	0.07 (0.14)	0.07 (0.15)	0.07 (0.17)	0.225 (0.251)	0.221 (0.247)	0.226 (0.252)	97.0	97.2	97.0	6.8	4.5	5.4	-115.6	-148.0	-128.6
	2.07	30	0.08 (0.18)	0.08 (0.19)	0.09 (0.18)	0.226 (0.256)	0.216 (0.250)	0.225 (0.253)	96.7	96.7	96.5	6.7	5.0	3.9	-112.6	-144.4	-127.9
	2.22	40	0.09 (0.17)	0.10 (0.20)	0.10 (0.18)	0.220 (0.253)	0.211 (0.249)	0.223 (0.254)	96.2	96.7	96.2	7.8	6.3	3.6	-108.5	-143.4	-131.8
	2.37	20	0.10 (0.20)	0.11 (0.21)	0.11 (0.21)	0.214 (0.243)	0.206 (0.244)	0.216 (0.240)	96.0	96.5	95.5	7.5	6.7	7.0	-109.5	-142.5	-122.1
	2.52	10	0.12 (0.22)	0.13 (0.23)	0.13 (0.22)	0.206 (0.251)	0.202 (0.243)	0.212 (0.248)	96.2	96.2	96.5	7.6	6.5	10.1	-109.1	-139.1	-120.6
	2.66	20	0.14 (0.24)	0.14 (0.25)	0.14 (0.26)	0.204 (0.244)	0.197 (0.244)	0.205 (0.244)	96.7	96.0	96.5	11.2	9.4	7.6	-105.5	-135.6	-122.2
	2.79	60	0.14 (0.26)	0.15 (0.30)	0.17 (0.26)	0.193 (0.245)	0.187 (0.251)	0.196 (0.245)	96.5	96.0	95.2	12.3	9.4	4.5	-104.0	-139.0	-134.0
	2.92	10	0.16 (0.28)	0.17 (0.27)	0.17 (0.28)	0.188 (0.230)	0.183 (0.235)	0.190 (0.230)	95.5	95.2	96.0	14.3	12.3	8.9	-104.3	-137.5	-118.4
	3.21	60	0.20 (0.38)	0.22 (0.39)	0.23 (0.34)	0.178 (0.248)	0.174 (0.255)	0.185 (0.240)	94.4	94.4	95.2	24.5	19.5	7.5	-89.3	-126.6	-141.0
	3.48	50	0.28 (0.50)	0.30 (0.47)	0.28 (0.49)	0.182 (0.258)	0.175 (0.259)	0.186 (0.251)	91.7	88.4	93.4	27.0	31.2	7.6	-83.6	-117.9	-137.3
	3.73	50	0.32 (0.57)	0.36 (0.51)	0.32 (0.52)	0.176 (0.260)	0.171 (0.267)	0.178 (0.255)	89.4	88.4	91.9	39.1	35.1	9.4	-65.8	-111.0	-135.8
	3.95	40	0.42 (0.67)	0.46 (0.58)	0.40 (0.63)	0.183 (0.301)	0.182 (0.314)	0.176 (0.278)	84.3	81.8	90.7	42.5	46.8	13.3	-62.9	-104.2	-124.7
1G8A (S7)	1.62	10	0.24 (0.28)	0.15 (0.28)	0.17 (0.40)	0.262 (0.279)	0.255 (0.277)	0.263 (0.279)	96.9	96.9	96.9	8.4	8.9	7.3	-46.3	-68.1	-46.3
	1.96	40	0.18 (0.32)	0.19 (0.42)	0.20 (0.35)	0.253 (0.280)	0.244 (0.273)	0.260 (0.282)	95.6	98.2	97.8	8.1	5.7	3.2	-46.9	-64.6	-60.3
	2.34	50	0.22 (0.40)	0.27 (0.42)	0.20 (0.39)	0.236 (0.310)	0.230 (0.307)	0.242 (0.303)	95.1	96.4	96.4	11.9	8.9	3.5	-39.0	-62.7	-70.6
	2.71	50	0.28 (0.46)	0.28 (0.51)	0.32 (0.47)	0.219 (0.295)	0.211 (0.296)	0.229 (0.286)	96.0	96.4	96.4	12.5	11.9	7.6	-41.5	-58.9	-61.0
	3.04	60	0.38 (0.59)	0.35 (0.61)	0.35 (0.60)	0.204 (0.297)	0.201 (0.315)	0.215 (0.296)	92.9	89.8	94.2	24.4	24.1	8.4	-29.3	-56.6	-69.5
	3.34	50	0.38 (0.72)	0.42 (0.69)	0.34 (0.65)	0.192 (0.313)	0.197 (0.323)	0.202 (0.289)	88.4	88.4	94.2	34.2	33.6	8.7	-26.6	-45.5	-64.6
	3.59	50	0.45 (0.76)	0.48 (0.76)	0.42 (0.71)	0.199 (0.357)	0.191 (0.329)	0.187 (0.302)	83.1	86.2	96.0	47.7	35.0	10.3	-16.2	-43.1	-68.0
	3.88	30	0.59 (1.00)	0.66 (0.83)	0.52 (0.93)	0.221 (0.372)	0.242 (0.406)	0.210 (0.337)	79.1	77.3	91.1	50.7	65.9	12.2	-12.7	-29.0	-54.0
	4.08	60	1.02 (1.49)	0.99 (0.93)	0.62 (1.44)	0.249 (0.375)	0.314 (0.450)	0.229 (0.325)	75.1	60.0	90.2	94.9	146.9	23.6	17.7	15.4	-71.1
	4.29	30	0.83 (1.21)	0.78 (1.11)	0.75 (1.18)	0.264 (0.414)	0.330 (0.471)	0.290 (0.398)	81.3	72.9	87.6	52.3	66.7	32.3	-4.8	-9.9	-47.2
	4.72	40	0.99 (1.22)	0.90 (1.18)	0.85 (1.35)	0.351 (0.455)	0.407 (0.410)	0.291 (0.417)	72.9	59.6	87.1	78.6	100.8	19.8	7.8	-12.2	-61.6
1G9O (S8)	1.67	10	0.08 (0.17)	0.08 (0.24)	0.08 (0.28)	0.261 (0.293)	0.262 (0.293)	0.264 (0.296)	100.0	98.9	98.9	6.3	3.5	1.4	-11.0	-14.9	-12.5
	1.92	10	0.10 (0.22)	0.10 (0.19)	0.10 (0.21)	0.254 (0.283)	0.255 (0.281)	0.255 (0.283)	100.0	100.0	100.0	5.6	3.5	1.4	-10.5	-14.7	-11.8
	2.19	10	0.13 (0.21)	0.12 (0.22)	0.13 (0.21)	0.247 (0.304)	0.248 (0.302)	0.248 (0.305)	98.9	98.9	98.9	8.4	7.0	3.5	-9.2	-13.7	-11.1
	2.44	10	0.16 (0.32)	0.17 (0.33)	0.17 (0.31)	0.234 (0.303)	0.234 (0.304)	0.235 (0.309)	98.9	98.9	98.9	3.5	4.2	1.4	-10.6	-15.1	-12.4
	2.64	10	0.18 (0.30)	0.18 (0.29)	0.17 (0.30)	0.221 (0.315)	0.220 (0.312)	0.224 (0.317)	97.8	98.9	98.9	9.8	6.3	3.5	-10.1	-14.7	-11.6
	2.87	20	0.21 (0.34)	0.22 (0.37)	0.23 (0.34)	0.206 (0.319)	0.209 (0.314)	0.214 (0.320)	100.0	100.0	97.8	10.5	9.8	4.2	-8.9	-14.3	-13.1
	3.10	50	0.24 (0.45)	0.26 (0.56)	0.30 (0.43)	0.204 (0.322)	0.208 (0.315)	0.231 (0.300)	95.5	97.8	97.8	11.9	9.1	2.8	-8.4	-13.1	-26.0
	3.20	10	0.28 (0.51)	0.31 (0.51)	0.33 (0.53)	0.221 (0.345)	0.211 (0.310)	0.225 (0.358)	91.0	94.4	93.3	12.6	14.0	11.9	-6.4	-13.1	-10.0
	3.33	20	0.30 (0.53)	0.33 (0.49)	0.30 (0.51)	0.203 (0.322)	0.198 (0.311)	0.208 (0.318)	93.3	92.1	93.3	14.7	14.7	6.3	-5.3	-14.0	-14.6
	3.48	50	0.39 (0.70)	0.43 (0.68)	0.35 (0.67)	0.213 (0.335)	0.210 (0.288)	0.217 (0.284)	84.3	92.1	89.9	23.2	32.3	4.2	-2.1	-10.1	-23.1
	3.94	60	0.60 (1.00)	0.62 (1.01)	0.56 (0.90)	0.254 (0.467)	0.224 (0.404)	0.213 (0.370)	79.8	75.3	78.7	57.5	56.1	9.8	3.2	-7.2	-22.7

PDB ID (ref)	Synth. Res. (Å)	w_{HINT}	RMSD vs. PDB (Å): Cα (all atom)			R (R_{free})			Ramachandran score			Clashscore			E_{HINT} (kcal mol^{-1})		
			0	0+e	0+H	0	0+e	0+H	0	0+e	0+H	0	0+e	0+H	0	0+e	0+H
1ZHV (S9)	1.69	50	0.23 (0.32)	0.23 (0.44)	0.22 (0.37)	0.266 (0.328)	0.266 (0.316)	0.273 (0.315)	97.0	96.2	97.0	4.4	5.4	0.0	-20.0	-26.7	-32.8
	1.85	10	0.21 (0.29)	0.21 (0.32)	0.21 (0.32)	0.260 (0.333)	0.259 (0.334)	0.261 (0.341)	97.7	96.2	97.7	6.9	7.4	4.4	-20.0	-25.9	-24.3
	2.03	20	0.23 (0.33)	0.21 (0.36)	0.22 (0.38)	0.253 (0.336)	0.255 (0.354)	0.257 (0.352)	97.7	97.0	97.0	4.4	5.9	1.5	-21.3	-26.1	-26.8
	2.27	60	0.26 (0.36)	0.23 (0.45)	0.24 (0.39)	0.258 (0.389)	0.254 (0.371)	0.269 (0.372)	97.0	96.2	96.2	6.9	9.9	3.0	-20.5	-25.4	-33.3
	2.49	50	0.26 (0.40)	0.23 (0.43)	0.25 (0.41)	0.242 (0.370)	0.236 (0.345)	0.246 (0.354)	93.9	93.9	97.0	5.9	7.9	0.5	-18.9	-25.1	-29.2
	2.69	10	0.28 (0.45)	0.30 (0.50)	0.29 (0.44)	0.216 (0.324)	0.216 (0.318)	0.227 (0.305)	93.2	92.4	94.7	11.8	12.3	4.4	-16.5	-23.1	-22.0
	2.89	30	0.32 (0.51)	0.32 (0.53)	0.33 (0.55)	0.220 (0.284)	0.214 (0.285)	0.223 (0.280)	85.6	88.6	96.2	21.7	15.3	3.0	-14.1	-23.1	-27.2
	3.06	40	0.39 (0.58)	0.37 (0.66)	0.47 (0.59)	0.234 (0.361)	0.248 (0.330)	0.241 (0.388)	90.2	93.9	92.4	13.3	14.3	6.4	-15.5	-22.0	-28.8
	3.25	10	0.47 (0.71)	0.51 (0.68)	0.50 (0.68)	0.214 (0.443)	0.215 (0.450)	0.224 (0.449)	79.5	84.1	83.3	30.6	24.7	21.2	-12.7	-22.4	-22.8
	3.50	20	0.67 (1.00)	0.79 (1.17)	0.83 (0.90)	0.372 (0.567)	0.336 (0.425)	0.416 (0.459)	72.0	83.3	80.3	76.5	33.0	23.7	-2.0	-18.9	-26.6
	3.76	10	0.64 (1.15)	0.77 (1.34)	1.05 (0.89)	0.319 (0.383)	0.277 (0.412)	0.317 (0.325)	63.6	75.0	72.0	88.8	60.7	46.9	12.4	-14.3	-13.0
1R7J (S10)	1.58	10	0.08 (0.22)	0.07 (0.23)	0.08 (0.27)	0.283 (0.313)	0.284 (0.313)	0.283 (0.312)	100.0	100.0	100.0	1.3	2.0	0.0	-21.0	-28.1	-24.3
	1.71	10	0.08 (0.25)	0.08 (0.25)	0.07 (0.27)	0.278 (0.301)	0.280 (0.301)	0.280 (0.299)	98.9	100.0	98.9	2.6	4.0	0.7	-19.8	-26.0	-23.4
	1.93	20	0.08 (0.30)	0.09 (0.29)	0.08 (0.31)	0.269 (0.299)	0.269 (0.298)	0.270 (0.292)	100.0	100.0	98.9	5.9	2.0	3.3	-20.2	-26.0	-25.2
	2.04	10	0.09 (0.27)	0.09 (0.28)	0.08 (0.31)	0.265 (0.290)	0.268 (0.290)	0.266 (0.293)	100.0	100.0	100.0	4.6	9.9	6.6	-20.0	-26.0	-24.1
	2.16	20	0.09 (0.33)	0.09 (0.34)	0.09 (0.32)	0.259 (0.309)	0.259 (0.315)	0.261 (0.310)	100.0	100.0	98.9	7.3	4.0	4.0	-19.5	-24.9	-24.5
	2.31	40	0.12 (0.33)	0.12 (0.37)	0.17 (0.33)	0.258 (0.303)	0.260 (0.305)	0.264 (0.303)	97.7	98.9	98.9	8.6	11.2	1.3	-20.1	-25.0	-29.7
	2.47	10	0.13 (0.38)	0.13 (0.37)	0.13 (0.38)	0.258 (0.313)	0.258 (0.310)	0.259 (0.316)	98.9	98.9	98.9	10.6	10.6	7.3	-18.8	-24.3	-22.2
	2.57	20	0.13 (0.37)	0.14 (0.40)	0.19 (0.39)	0.250 (0.302)	0.251 (0.287)	0.254 (0.300)	98.9	98.9	98.9	12.6	9.9	8.6	-18.9	-25.1	-24.8
	2.70	20	0.14 (0.37)	0.14 (0.36)	0.16 (0.35)	0.251 (0.314)	0.252 (0.314)	0.253 (0.314)	97.7	97.7	98.9	11.9	9.2	6.6	-19.9	-25.6	-24.7
	2.83	60	0.16 (0.36)	0.16 (0.44)	0.18 (0.39)	0.243 (0.339)	0.244 (0.340)	0.248 (0.325)	97.7	98.9	97.7	11.9	10.6	4.6	-19.3	-24.3	-29.8
	3.24	60	0.26 (0.64)	0.30 (0.61)	0.28 (0.61)	0.242 (0.338)	0.242 (0.342)	0.245 (0.325)	92.0	95.5	95.5	23.8	28.4	3.3	-14.3	-18.2	-31.6
	3.59	20	0.36 (0.68)	0.36 (0.65)	0.33 (0.68)	0.223 (0.293)	0.232 (0.312)	0.226 (0.297)	93.2	93.2	93.2	34.4	31.7	11.2	-10.5	-17.4	-24.1
	3.96	50	0.43 (0.77)	0.41 (0.74)	0.39 (0.75)	0.266 (0.391)	0.257 (0.393)	0.254 (0.376)	87.5	92.0	89.8	22.5	33.7	2.6	-12.0	-22.4	-31.3
	4.20	60	0.58 (1.13)	0.70 (0.92)	0.51 (0.97)	0.351 (0.480)	0.314 (0.398)	0.317 (0.373)	69.3	79.5	90.9	44.2	48.9	8.6	-7.5	-15.3	-31.7
1UOY (S11)	1.68	10	0.07 (0.10)	0.07 (0.14)	0.08 (0.16)	0.272 (0.296)	0.273 (0.292)	0.273 (0.292)	95.2	95.2	95.2	2.3	5.8	2.3	-17.2	-22.7	-20.7
	2.13	20	0.11 (0.15)	0.11 (0.21)	0.12 (0.16)	0.271 (0.293)	0.269 (0.283)	0.275 (0.291)	96.8	96.8	98.4	7.0	8.2	3.5	-16.2	-21.7	-21.3
	2.45	40	0.14 (0.19)	0.15 (0.24)	0.15 (0.22)	0.262 (0.331)	0.261 (0.325)	0.274 (0.314)	96.8	96.8	91.9	7.0	11.7	1.2	-15.6	-22.8	-22.6
	2.72	20	0.19 (0.30)	0.21 (0.30)	0.20 (0.26)	0.264 (0.331)	0.260 (0.305)	0.264 (0.332)	93.5	93.5	90.3	17.5	16.4	7.0	-13.2	-20.4	-20.1
	3.11	20	0.26 (0.37)	0.25 (0.40)	0.26 (0.38)	0.237 (0.344)	0.242 (0.335)	0.248 (0.329)	91.9	90.3	91.9	26.9	22.2	7.0	-13.3	-19.9	-19.9
	3.29	20	0.33 (0.54)	0.36 (0.45)	0.34 (0.47)	0.277 (0.376)	0.265 (0.351)	0.286 (0.352)	93.5	93.5	85.5	39.8	24.6	16.4	-9.9	-20.0	-17.9
	3.58	30	0.36 (0.78)	0.60 (0.65)	0.45 (0.58)	0.331 (0.426)	0.267 (0.372)	0.296 (0.394)	75.8	87.1	85.5	29.2	28.1	15.2	-10.0	-17.8	-23.1
	3.78	40	0.42 (0.69)	0.49 (0.76)	0.52 (0.62)	0.265 (0.405)	0.256 (0.352)	0.283 (0.413)	77.4	87.1	79.0	32.8	43.3	10.5	-9.8	-17.0	-23.9
	4.01	40	0.48 (0.69)	0.51 (0.71)	0.52 (0.66)	0.284 (0.346)	0.292 (0.384)	0.286 (0.349)	88.7	87.1	83.9	31.6	32.8	12.9	-8.5	-15.6	-24.1
1RL0 (S12)	1.70	10	0.09 (0.25)	0.08 (0.32)	0.08 (0.28)	0.255 (0.286)	0.257 (0.284)	0.258 (0.289)	97.6	98.0	97.6	2.7	2.7	1.2	-65.4	-88.5	-80.6
	2.12	10	0.12 (0.30)	0.12 (0.30)	0.12 (0.30)	0.242 (0.285)	0.243 (0.285)	0.244 (0.286)	96.8	97.6	97.6	3.5	3.0	2.5	-63.6	-83.2	-78.5
	2.33	20	0.14 (0.35)	0.14 (0.38)	0.13 (0.37)	0.237 (0.285)	0.240 (0.293)	0.239 (0.289)	97.6	96.8	98.0	3.9	5.2	1.5	-61.5	-85.5	-83.7
	2.42	50	0.15 (0.33)	0.15 (0.39)	0.16 (0.35)	0.232 (0.297)	0.232 (0.294)	0.254 (0.291)	98.0	98.0	97.6	5.7	3.9	2.7	-60.6	-82.7	-91.6
	2.58	30	0.17 (0.36)	0.17 (0.36)	0.16 (0.37)	0.224 (0.294)	0.225 (0.295)	0.227 (0.294)	95.3	96.4	97.2	5.9	8.4	1.5	-61.1	-80.8	-85.8
	2.73	10	0.19 (0.39)	0.19 (0.38)	0.18 (0.40)	0.211 (0.280)	0.213 (0.273)	0.213 (0.275)	94.9	96.0	96.8	7.1	6.7	3.2	-62.5	-85.7	-77.7
	2.87	30	0.20 (0.40)	0.20 (0.41)	0.19 (0.41)	0.207 (0.309)	0.207 (0.305)	0.208 (0.307)	94.9	96.4	95.3	8.1	9.4	1.7	-62.4	-83.2	-87.1
	2.95	40	0.22 (0.45)	0.22 (0.45)	0.22 (0.46)	0.196 (0.319)	0.198 (0.319)	0.200 (0.306)	94.9	94.9	96.4	10.1	10.1	2.2	-56.3	-75.1	-88.1
	3.06	50	0.24 (0.44)	0.24 (0.53)	0.23 (0.45)	0.194 (0.308)	0.197 (0.311)	0.210 (0.297)	93.7	93.3	96.0	11.8	13.8	3.0	-55.9	-78.8	-94.1
	3.18	10	0.28 (0.51)	0.29 (0.49)	0.27 (0.52)	0.185 (0.310)	0.188 (0.309)	0.185 (0.302)	90.5	92.1	90.5	17.0	17.7	10.1	-50.5	-73.7	-68.6
	3.52	30	0.35 (0.66)	0.40 (0.62)	0.35 (0.60)	0.176 (0.301)	0.175 (0.289)	0.176 (0.291)	87.7	87.7	92.1	23.1	19.5	5.2	-42.3	-73.4	-84.1
	3.94	40	0.53 (0.89)	0.55 (0.75)	0.48 (0.87)	0.190 (0.340)	0.197 (0.341)	0.184 (0.322)	73.5	75.5	86.6	45.8	48.8	8.9	-21.5	-48.0	-85.9
	4.14	60	0.57 (0.90)	0.61 (0.80)	0.54 (0.87)	0.186 (0.361)	0.177 (0.332)	0.191 (0.327)	74.7	83.8	88.9	43.6	37.4	5.4	-22.4	-46.2	-100.8
	4.31	60	0.61 (0.93)	0.59 (0.87)	0.52 (0.92)	0.190 (0.344)	0.184 (0.333)	0.173 (0.303)	76.7	79.4	86.2	31.8	41.1	7.9	-18.9	-56.5	-111.6
1TUA (S13)	1.69	10	0.08 (0.26)	0.07 (0.36)	0.07 (0.42)	0.232 (0.333)	0.235 (0.336)	0.234 (0.332)	98.9	98.9	98.9	4.2	4.9	1.6	-40.5	-56.4	-47.3
	1.85	20	0.08 (0.26)	0.07 (0.31)	0.12 (0.40)	0.226 (0.316)	0.228 (0.301)	0.228 (0.303)	97.9	97.9	98.9	3.9	5.8	1.0	-38.9	-56.8	-52.0
	1.98	10	0.09 (0.26)	0.07 (0.27)	0.08 (0.34)	0.219 (0.264)	0.223 (0.283)	0.221 (0.269)	98.9	98.9	98.4	1.9	4.2	1.6	-39.9	-53.5	-45.9
	2.13	20	0.11 (0.28)	0.09 (0.31)	0.11 (0.41)	0.216 (0.299)	0.221 (0.304)	0.218 (0.295)	98.9	98.9	98.4	4.5	7.1	1.3	-39.5	-55.2	-50.4
	2.29	10	0.10 (0.29)	0.09 (0.30)	0.10 (0.34)	0.210 (0.280)	0.212 (0.279)	0.212 (0.272)	98.9	99.5	98.9	4.5	6.2	2.6	-38.7	-50.4	-45.0

PDB ID (ref)	Synth. Res. (Å)	w_{HINT}	RMSD vs. PDB (Å): Cα (all atom)			R (R_{free})			Ramachandran score			Clashscore			E_{HINT} (kcal mol^{-1})		
			0	0+e	0+H	0	0+e	0+H	0	0+e	0+H	0	0+e	0+H	0	0+e	0+H
1TUA (cont.)	2.46	10	0.12 (0.34)	0.12 (0.35)	0.12 (0.40)	0.211 (0.265)	0.211 (0.277)	0.215 (0.265)	98.4	98.4	98.4	3.9	5.8	3.2	-38.8	-49.7	-44.5
	2.62	20	0.14 (0.32)	0.12 (0.35)	0.14 (0.40)	0.202 (0.254)	0.204 (0.253)	0.205 (0.257)	97.9	98.4	97.9	6.8	6.5	1.9	-39.4	-53.4	-48.5
	2.75	50	0.17 (0.41)	0.19 (0.66)	0.41 (0.42)	0.213 (0.354)	0.210 (0.349)	0.223 (0.319)	95.7	97.9	95.2	13.6	10.1	3.6	-34.3	-49.1	-63.8
	2.88	10	0.17 (0.38)	0.17 (0.38)	0.16 (0.43)	0.199 (0.268)	0.203 (0.295)	0.201 (0.269)	97.3	96.8	96.8	7.8	12.0	5.2	-36.7	-50.3	-43.2
	3.00	20	0.18 (0.41)	0.19 (0.43)	0.18 (0.46)	0.205 (0.287)	0.209 (0.313	0.208 (0.273)	95.7	96.8	96.8	11.0	9.4	3.2	-36.5	-49.5	-47.8
	3.26	30	0.30 (0.61)	0.30 (0.63)	0.29 (0.63)	0.204 (0.428)	0.215 (0.439)	0.213 (0.381)	91.4	94.7	95.7	20.4	22.1	4.5	-24.5	-37.9	-55.3
	3.52	30	0.55 (0.92)	0.55 (0.71)	0.40 (0.89)	0.278 (0.538)	0.276 (0.512)	0.254 (0.458)	76.5	79.7	88.2	53.2	50.9	7.8	-7.7	-26.3	-43.2
	3.79	10	0.65 (1.10)	0.71 (1.02)	0.64 (1.04)	0.352 (0.578)	0.341 (0.540	0.359 (0.595)	65.8	82.9	74.9	79.8	54.1	41.2	8.0	-18.6	-16.7
1VDQ (S14)	1.68	10	0.07 (0.29)	0.06 (0.39)	0.07 (0.34)	0.220 (0.241)	0.222 (0.243)	0.221 (0.241)	100.0	99.2	99.2	2.0	3.6	0.5	-34.0	-44.1	-40.0
	1.85	10	0.07 (0.31)	0.07 (0.39)	0.08 (0.34)	0.215 (0.241)	0.217 (0.239)	0.216 (0.240)	99.2	99.2	99.2	2.0	3.6	1.5	-34.5	-43.4	-39.9
	2.07	30	0.10 (0.36)	0.09 (0.41)	0.12 (0.38)	0.210 (0.244)	0.212 (0.242)	0.216 (0.240)	100.0	99.2	98.4	3.1	3.6	1.5	-34.2	-42.8	-43.1
	2.33	10	0.11 (0.42)	0.11 (0.42)	0.11 (0.37)	0.199 (0.236)	0.199 (0.235)	0.200 (0.234)	98.4	97.6	98.4	3.1	3.1	2.0	-34.5	-42.3	-38.5
	2.59	20	0.13 (0.41)	0.14 (0.51)	0.15 (0.43)	0.190 (0.256)	0.191 (0.253)	0.196 (0.258)	98.4	96.9	96.9	4.1	3.6	3.1	-31.7	-41.7	-42.4
	2.79	10	0.15 (0.48)	0.15 (0.47)	0.16 (0.46)	0.177 (0.262)	0.177 (0.267)	0.178 (0.260)	96.1	94.5	95.3	6.7	8.2	4.6	-32.5	-41.7	-38.3
	3.02	20	0.20 (0.52)	0.20 (0.57)	0.20 (0.54)	0.170 (0.269)	0.170 (0.273)	0.170 (0.265)	96.1	95.3	96.1	6.2	10.3	2.6	-29.7	-38.8	-43.0
	3.23	10	0.24 (0.61)	0.29 (0.59)	0.25 (0.53)	0.165 (0.284)	0.168 (0.283)	0.169 (0.271)	92.9	94.5	92.1	9.2	10.8	8.2	-24.6	-36.7	-36.1
	3.39	40	0.29 (0.65)	0.34 (0.67)	0.27 (0.53)	0.165 (0.287)	0.164 (0.262)	0.163 (0.256)	89.8	93.7	92.9	17.9	16.9	4.1	-24.1	-38.5	-47.9
	3.59	10	0.34 (0.66)	0.33 (0.63)	0.30 (0.59)	0.145 (0.297)	0.148 (0.304)	0.152 (0.286)	85.0	89.0	89.0	22.1	27.2	11.3	-23.4	-37.8	-36.8
	4.01	20	0.48 (0.90)	0.55 (0.81)	0.47 (0.76)	0.215 (0.373)	0.193 (0.340)	0.196 (0.335)	72.4	79.5	82.7	38.5	32.8	19.0	-12.0	-27.3	-39.2
1YU5 (S15)	1.68	10	0.09 (0.17)	0.07 (0.15)	0.08 (0.19)	0.281 (0.306)	0.283 (0.306)	0.285 (0.307)	96.9	96.9	96.9	1.9	0.0	2.8	-14.7	-16.9	-15.6
	1.93	20	0.11 (0.24)	0.09 (0.24)	0.11 (0.23)	0.273 (0.313)	0.275 (0.314)	0.279 (0.315)	98.5	98.5	96.9	0.0	0.9	0.0	-14.4	-16.8	-16.3
	2.14	20	0.13 (0.26)	0.13 (0.28)	0.14 (0.26)	0.266 (0.326)	0.267 (0.323)	0.272 (0.324)	96.9	96.9	96.9	3.7	3.7	1.9	-13.6	-15.6	-15.3
	2.40	30	0.19 (0.37)	0.20 (0.46)	0.18 (0.33)	0.262 (0.375)	0.263 (0.366)	0.277 (0.379)	96.9	96.9	98.5	5.6	1.9	0.9	-12.5	-14.7	-15.5
	2.61	30	0.21 (0.35)	0.21 (0.42)	0.20 (0.36)	0.240 (0.365)	0.242 (0.365)	0.253 (0.365)	98.5	95.4	98.5	5.6	6.5	0.9	-13.2	-15.5	-18.3
	2.74	10	0.24 (0.48)	0.24 (0.42)	0.25 (0.48)	0.250 (0.396)	0.247 (0.367)	0.242 (0.352)	95.4	93.8	93.8	19.5	18.6	14.8	-9.2	-12.7	-11.6
	2.80	20	0.25 (0.47)	0.27 (0.42)	0.24 (0.44)	0.241 (0.335)	0.236 (0.337)	0.242 (0.339)	93.8	95.4	95.4	8.3	8.3	1.9	-12.8	-15.6	-16.9
	2.99	40	0.28 (0.51)	0.29 (0.56)	0.27 (0.50)	0.238 (0.393)	0.230 (0.386)	0.235 (0.347)	92.3	90.8	95.4	15.8	17.6	4.6	-10.0	-13.9	-17.8
	3.60	50	0.45 (0.76)	0.47 (0.77)	0.44 (0.77)	0.233 (0.359)	0.222 (0.329)	0.229 (0.304)	87.7	93.8	87.7	33.4	39.0	9.3	-9.1	-9.9	-22.2
	3.68	50	0.47 (1.03)	0.69 (1.02)	0.56 (0.83)	0.287 (0.409)	0.286 (0.448)	0.270 (0.340)	75.4	84.6	81.5	58.4	31.5	9.3	-1.7	-13.4	-19.2
	3.75	60	0.81 (1.01)	0.70 (1.16)	0.82 (1.07)	0.285 (0.445)	0.314 (0.451)	0.320 (0.439)	69.2	76.9	70.8	41.7	52.0	14.8	-4.9	-8.8	-14.2
	4.08	50	0.70 (1.25)	0.92 (1.12)	0.70 (1.06)	0.294 (0.481)	0.272 (0.394)	0.256 (0.382)	72.3	80.0	78.5	65.9	40.8	13.9	0.1	-10.9	-17.2
3KJT (S16)	1.71	10	0.08 (0.33)	0.08 (0.35)	0.07 (0.39)	0.240 (0.267)	0.241 (0.270)	0.240 (0.268)	98.4	98.1	98.4	9.9	9.6	5.9	-72.2	-107.7	-97.7
	1.87	10	0.09 (0.34)	0.08 (0.34)	0.08 (0.38)	0.235 (0.264)	0.237 (0.267)	0.237 (0.263)	98.1	97.6	97.6	8.9	8.4	5.8	-72.3	-102.3	-93.7
	1.99	20	0.11 (0.37)	0.10 (0.40)	0.12 (0.43)	0.230 (0.274)	0.231 (0.271)	0.232 (0.273)	97.0	97.3	97.6	8.5	10.1	4.9	-74.1	-100.7	-103.0
	2.11	50	0.13 (0.40)	0.12 (0.51)	0.15 (0.44)	0.227 (0.275)	0.227 (0.274)	0.236 (0.275)	97.8	97.6	97.3	8.9	9.4	3.7	-73.8	-102.7	-124.3
	2.25	10	0.13 (0.45)	0.13 (0.44)	0.13 (0.47)	0.219 (0.272)	0.220 (0.269)	0.218 (0.266)	97.3	97.3	97.6	8.9	8.7	6.8	-65.0	-98.1	-87.2
	2.40	30	0.16 (0.46)	0.16 (0.45)	0.18 (0.50)	0.210 (0.275)	0.211 (0.272)	0.215 (0.272)	97.0	96.5	97.3	9.2	10.6	4.9	-71.4	-93.7	-104.3
	2.54	20	0.17 (0.49)	0.17 (0.47)	0.17 (0.53)	0.207 (0.280)	0.211 (0.288)	0.212 (0.281)	95.7	95.9	95.9	13.4	15.5	5.6	-66.5	-91.8	-94.6
	2.64	40	0.18 (0.48)	0.18 (0.54)	0.20 (0.50)	0.201 (0.300)	0.203 (0.293)	0.209 (0.285)	95.1	96.5	95.7	13.6	14.6	4.7	-65.0	-92.1	-109.4
	2.78	30	0.20 (0.50)	0.21 (0.49)	0.20 (0.50)	0.192 (0.284)	0.194 (0.282)	0.200 (0.287)	94.0	95.4	95.1	16.0	17.4	5.9	-61.9	-88.2	-101.2
	2.89	30	0.23 (0.50)	0.23 (0.50)	0.21 (0.54)	0.186 (0.292)	0.189 (0.287)	0.191 (0.283)	95.9	95.9	95.9	13.4	15.9	5.9	-64.8	-96.1	-103.6
	3.13	60	0.30 (0.62)	0.31 (0.66)	0.32 (0.62)	0.182 (0.310)	0.181 (0.304)	0.191 (0.300)	92.4	91.8	92.7	21.1	22.0	5.6	-53.9	-85.0	-124.9
	3.28	60	0.36 (0.74)	0.40 (0.73)	0.37 (0.71)	0.193 (0.348)	0.186 (0.317)	0.191 (0.314)	83.2	89.7	91.6	37.6	31.9	8.2	-44.3	-76.4	-129.2
	3.52	50	0.40 (0.76)	0.42 (0.77)	0.40 (0.77)	0.200 (0.352)	0.191 (0.346)	0.194 (0.329)	84.8	86.7	91.8	39.7	38.7	10.4	-38.0	-72.4	-117.8
	3.73	40	0.57 (0.93)	0.60 (0.84)	0.52 (0.91)	0.213 (0.357)	0.200 (0.342)	0.200 (0.338)	77.7	84.2	87.2	49.1	48.1	11.5	-18.5	-54.3	-106.9
2P4H (S17)	1.60	10	0.65 (0.38)	0.34 (0.47)	0.40 (0.70)	0.257 (0.277)	0.258 (0.278)	0.259 (0.280)	97.0	97.0	96.4	3.1	3.1	2.5	-80.3	-103.7	-92.4
	1.83	20	0.12 (0.41)	0.38 (0.40)	0.36 (0.39)	0.256 (0.282)	0.255 (0.282)	0.258 (0.283)	97.0	97.7	97.4	4.3	3.9	2.3	-78.1	-106.0	-97.5
	2.07	10	0.34 (0.32)	0.20 (0.39)	0.34 (0.41)	0.250 (0.275)	0.252 (0.279)	0.253 (0.277)	97.4	96.7	97.7	4.8	5.4	2.7	-77.5	-104.8	-88.9
	2.33	20	0.17 (0.52)	0.33 (0.36)	0.25 (0.45)	0.244 (0.300)	0.245 (0.295)	0.246 (0.294)	95.7	96.4	96.7	5.8	4.8	2.5	-75.0	-102.4	-94.5
	2.45	30	0.15 (0.38)	0.18 (0.42)	0.22 (0.37)	0.239 (0.291)	0.241 (0.291)	0.243 (0.285)	97.4	95.4	97.0	6.0	6.4	1.4	-72.1	-95.4	-99.3
	2.66	40	0.23 (0.41)	0.27 (0.45)	0.26 (0.41)	0.230 (0.303)	0.227 (0.301)	0.235 (0.304)	96.4	96.1	95.7	8.7	7.4	1.2	-72.6	-94.2	-107.5
	2.87	40	0.31 (0.40)	0.26 (0.45)	0.30 (0.42)	0.213 (0.299)	0.215 (0.302)	0.219 (0.287)	95.4	95.4	94.4	12.0	14.3	2.9	-67.2	-93.2	-103.1
	2.96	10	0.28 (0.43)	0.25 (0.48)	0.27 (0.46)	0.205 (0.296)	0.207 (0.299)	0.209 (0.292)	93.4	93.4	94.1	14.3	13.0	7.9	-62.8	-87.3	-81.4

PDB ID (ref)	Synth. Res. (Å)	w_{HINT}	RMSD vs. PDB (Å): Cα (all atom)			R (R_{free})			Ramachandran score			Clashscore			E_{HINT} (kcal mol^{-1})		
			0	0+e	0+H	0	0+e	0+H	0	0+e	0+H	0	0+e	0+H	0	0+e	0+H
2P4H (cont.)	3.08	50	0.27 (0.44)	0.28 (0.48)	0.31 (0.43)	0.208 (0.305)	0.203 (0.298)	0.214 (0.298)	92.8	92.4	93.4	16.5	12.8	4.1	-64.5	-94.2	-111.0
	3.23	50	0.37 (0.68)	0.44 (0.64)	0.44 (0.62)	0.212 (0.333)	0.202 (0.319)	0.213 (0.315)	85.5	86.2	89.1	31.8	35.0	7.4	-49.6	-73.9	-109.7
	3.65	50	0.45 (0.78)	0.46 (0.70)	0.42 (0.75)	0.194 (0.338)	0.195 (0.342)	0.201 (0.326)	78.0	79.3	84.2	50.7	41.6	9.1	-38.7	-57.0	-109.7
	4.00	30	0.66 (1.02)	0.70 (0.84)	0.56 (0.97)	0.249 (0.376)	0.247 (0.376)	0.224 (0.361)	68.4	78.0	82.9	71.5	66.2	18.8	-21.3	-59.5	-95.0
	4.34	30	0.70 (1.09)	0.73 (0.95)	0.68 (1.05)	0.213 (0.403)	0.221 (0.373)	0.198 (0.331)	66.8	72.0	81.9	69.7	70.7	20.9	-22.0	-54.8	-99.4
	4.61	50	0.84 (1.19)	0.82 (1.04)	0.75 (1.23)	0.238 (0.428)	0.227 (0.427)	0.220 (0.409)	68.8	68.4	79.3	59.8	70.5	13.0	-18.4	-50.8	-114.3
3L83 (S18)	1.93	10	0.08 (0.17)	0.08 (0.17)	0.09 (0.19)	0.268 (0.305)	0.270 (0.307)	0.266 (0.306)	97.0	97.0	97.4	3.5	4.1	2.5	-48.8	-55.6	-55.9
	2.28	10	0.11 (0.21)	0.11 (0.21)	0.11 (0.22)	0.256 (0.299)	0.256 (0.299)	0.258 (0.299)	97.0	97.0	96.6	5.2	6.0	4.1	-46.7	-53.5	-53.0
	2.68	40	0.16 (0.34)	0.20 (0.35)	0.20 (0.29)	0.237 (0.336)	0.239 (0.332)	0.241 (0.325)	96.6	96.2	97.0	12.3	13.9	2.7	-40.9	-48.9	-64.3
	2.81	20	0.17 (0.32)	0.17 (0.30)	0.17 (0.31)	0.231 (0.296)	0.231 (0.293)	0.232 (0.290)	96.6	97.0	97.0	15.8	17.4	6.0	-39.7	-48.2	-51.9
	3.10	60	0.22 (0.41)	0.23 (0.38)	0.23 (0.40)	0.206 (0.296)	0.201 (0.291)	0.212 (0.284)	93.6	94.0	94.0	22.6	21.8	8.2	-33.0	-42.0	-62.9
	3.51	60	0.43 (0.68)	0.46 (0.58)	0.37 (0.65)	0.204 (0.331)	0.202 (0.334)	0.205 (0.325)	80.4	85.1	89.4	49.9	42.3	12.3	-16.6	-33.2	-69.5
	3.71	10	0.35 (0.66)	0.44 (0.53)	0.33 (0.57)	0.186 (0.311)	0.185 (0.286)	0.186 (0.283)	80.9	91.1	90.6	50.2	50.7	32.7	-23.7	-29.9	-35.1
	3.90	50	0.42 (0.66)	0.41 (0.53)	0.34 (0.67)	0.181 (0.305)	0.183 (0.300)	0.181 (0.267)	87.2	85.5	93.2	58.1	55.4	13.9	-22.6	-31.6	-63.0
	4.11	50	0.51 (0.78)	0.51 (0.60)	0.38 (0.77)	0.204 (0.344)	0.204 (0.338)	0.196 (0.320)	81.7	83.0	91.5	75.0	76.6	22.4	-7.1	-20.2	-59.8
	4.33	60	0.62 (0.97)	0.67 (0.70)	0.45 (0.89)	0.210 (0.417)	0.196 (0.390)	0.203 (0.366)	69.8	74.0	87.2	86.2	72.3	16.1	1.8	-12.4	-63.0
1WPA (S19)	1.69	20	0.11 (0.26)	0.09 (0.33)	0.12 (0.28)	0.273 (0.304)	0.274 (0.301)	0.277 (0.304)	100.0	100.0	100.0	3.9	5.6	2.8	-23.5	-31.2	-33.3
	1.88	10	0.11 (0.29)	0.10 (0.29)	0.12 (0.34)	0.264 (0.299)	0.264 (0.293)	0.267 (0.296)	100.0	100.0	100.0	5.0	2.8	2.2	-21.6	-31.6	-29.2
	2.03	30	0.12 (0.28)	0.11 (0.45)	0.18 (0.33)	0.256 (0.310)	0.259 (0.308)	0.262 (0.307)	100.0	100.0	100.0	4.5	5.6	2.2	-23.8	-31.7	-34.1
	2.21	10	0.12 (0.38)	0.12 (0.40)	0.14 (0.40)	0.251 (0.316)	0.252 (0.314)	0.250 (0.313)	100.0	99.0	100.0	5.0	5.0	1.7	-22.3	-29.2	-27.5
	2.45	40	0.14 (0.40)	0.14 (0.47)	0.22 (0.43)	0.243 (0.324)	0.249 (0.335)	0.253 (0.316)	100.0	100.0	100.0	6.1	7.2	0.6	-22.8	-30.5	-38.0
	2.74	10	0.19 (0.49)	0.22 (0.49)	0.20 (0.51)	0.232 (0.313)	0.238 (0.310)	0.232 (0.316)	99.0	99.0	100.0	8.3	6.7	2.8	-21.7	-33.1	-28.8
	2.89	10	0.20 (0.49)	0.20 (0.53)	0.19 (0.53)	0.223 (0.327)	0.233 (0.332)	0.223 (0.331)	99.0	98.1	100.0	8.3	9.4	4.5	-22.2	-31.6	-30.7
	3.05	30	0.23 (0.46)	0.23 (0.54)	0.24 (0.51)	0.228 (0.330)	0.229 (0.343)	0.238 (0.329)	100.0	100.0	98.1	10.6	13.3	2.2	-20.8	-32.9	-34.9
	3.21	30	0.30 (0.67)	0.31 (0.77)	0.29 (0.68)	0.239 (0.367)	0.253 (0.379)	0.248 (0.349)	92.4	90.5	96.2	20.6	29.5	5.6	-20.0	-30.2	-37.4
	3.34	20	0.42 (0.86)	0.39 (0.77)	0.40 (0.81)	0.255 (0.399)	0.261 (0.393)	0.250 (0.364)	84.8	86.7	90.5	35.6	47.2	10.6	-15.7	-29.5	-35.6
	3.67	40	0.50 (0.91)	0.48 (0.97)	0.49 (0.89)	0.259 (0.422)	0.231 (0.400)	0.250 (0.383)	85.7	91.4	89.5	53.4	50.0	12.2	-9.6	-25.9	-41.6
	3.94	50	0.56 (0.98)	0.59 (1.13)	0.68 (0.95)	0.243 (0.395)	0.265 (0.431)	0.259 (0.382)	81.0	81.0	91.4	34.5	46.1	16.1	-12.4	-30.5	-34.0
	4.23	30	0.63 (1.09)	0.69 (1.04)	0.60 (1.05)	0.344 (0.552)	0.292 (0.468)	0.307 (0.464)	73.3	81.9	84.8	60.6	51.1	22.8	-7.7	-22.3	-35.8
3FE0 (S20)	1.70	10	0.06 (0.17)	0.05 (0.30)	0.05 (0.25)	0.227 (0.257)	0.229 (0.261)	0.231 (0.259)	99.2	100.0	99.2	1.5	2.5	1.0	-38.7	-46.2	-43.1
	1.96	10	0.07 (0.19)	0.07 (0.34)	0.07 (0.23)	0.218 (0.259)	0.221 (0.257)	0.221 (0.262)	100.0	100.0	100.0	2.5	3.5	3.0	-38.1	-45.5	-43.5
	2.20	40	0.10 (0.26)	0.10 (0.30)	0.11 (0.31)	0.215 (0.278)	0.219 (0.272)	0.222 (0.265)	99.2	99.2	99.2	2.0	2.5	1.0	-37.0	-45.7	-46.8
	2.46	20	0.13 (0.30)	0.13 (0.30)	0.13 (0.29)	0.201 (0.252)	0.205 (0.253)	0.205 (0.255)	98.4	98.4	98.4	4.0	3.5	2.0	-36.0	-44.7	-42.5
	2.70	60	0.14 (0.29)	0.14 (0.35)	0.15 (0.31)	0.187 (0.253)	0.191 (0.257)	0.204 (0.248)	98.4	97.7	97.7	4.0	5.9	3.5	-35.3	-45.3	-49.8
	2.87	10	0.17 (0.38)	0.18 (0.38)	0.17 (0.39)	0.183 (0.280)	0.186 (0.278)	0.184 (0.277)	94.5	96.9	95.3	10.4	7.9	5.9	-32.6	-44.5	-38.3
	3.06	10	0.22 (0.41)	0.22 (0.41)	0.21 (0.42)	0.178 (0.290)	0.180 (0.285)	0.179 (0.290)	93.8	96.9	93.8	8.9	10.9	8.4	-34.4	-44.2	-38.6
	3.18	30	0.23 (0.49)	0.26 (0.45)	0.23 (0.47)	0.187 (0.342)	0.186 (0.326)	0.183 (0.307)	96.1	95.3	94.5	21.3	14.3	7.4	-29.7	-40.9	-44.9
	3.34	20	0.32 (0.61)	0.34 (0.55)	0.30 (0.59)	0.192 (0.345)	0.183 (0.303)	0.181 (0.291)	85.2	87.5	86.7	24.7	25.2	15.3	-25.1	-37.6	-41.0
	3.51	50	0.31 (0.56)	0.33 (0.51)	0.28 (0.55)	0.177 (0.315)	0.169 (0.291)	0.168 (0.263)	91.4	85.9	93.8	21.8	29.2	5.9	-28.3	-36.5	-50.8
	3.99	50	0.47 (0.80)	0.52 (0.64)	0.39 (0.70)	0.202 (0.374)	0.190 (0.384)	0.187 (0.360)	71.9	83.6	82.8	47.0	34.1	10.4	-16.5	-33.0	-51.2
2FQ3 (S21)	1.55	10	0.05 (0.24)	0.05 (0.31)	0.06 (0.27)	0.254 (0.274)	0.257 (0.279)	0.259 (0.281)	97.6	98.8	97.6	1.4	3.5	1.4	-18.5	-21.0	-21.4
	1.71	10	0.06 (0.27)	0.07 (0.30)	0.07 (0.29)	0.249 (0.292)	0.251 (0.294)	0.250 (0.292)	98.8	98.8	98.8	4.2	4.9	1.4	-18.0	-22.7	-21.4
	1.96	40	0.09 (0.28)	0.09 (0.30)	0.12 (0.32)	0.240 (0.276)	0.240 (0.277)	0.244 (0.273)	98.8	98.8	98.8	2.8	3.5	1.4	-17.6	-20.7	-23.6
	2.29	10	0.11 (0.25)	0.11 (0.31)	0.12 (0.29)	0.234 (0.281)	0.235 (0.277)	0.233 (0.279)	98.8	98.8	98.8	3.5	7.0	2.1	-17.7	-21.7	-20.7
	2.44	30	0.12 (0.29)	0.12 (0.30)	0.14 (0.29)	0.233 (0.295)	0.233 (0.293)	0.234 (0.287)	98.8	97.6	97.6	7.0	6.2	4.2	-16.7	-21.7	-21.7
	2.67	40	0.16 (0.39)	0.15 (0.37)	0.17 (0.40)	0.219 (0.322)	0.220 (0.323)	0.226 (0.316)	97.6	97.6	96.4	7.0	4.2	1.4	-17.3	-20.2	-23.1
	2.81	10	0.19 (0.36)	0.18 (0.39)	0.18 (0.37)	0.215 (0.287)	0.217 (0.282)	0.220 (0.289)	98.8	98.8	98.8	4.9	7.0	4.2	-15.9	-19.4	-18.5
	2.92	10	0.19 (0.41)	0.19 (0.40)	0.19 (0.35)	0.204 (0.312)	0.205 (0.316)	0.206 (0.309)	98.8	98.8	98.8	6.2	6.2	2.8	-16.1	-20.4	-18.9
	3.64	40	0.38 (0.82)	0.41 (0.69)	0.38 (0.68)	0.209 (0.359)	0.216 (0.338)	0.232 (0.329)	79.5	85.5	97.6	25.0	21.5	3.5	-14.0	-21.9	-25.8
	3.76	40	0.57 (0.96)	0.61 (0.83)	0.48 (0.94)	0.266 (0.522)	0.261 (0.457)	0.247 (0.395)	65.1	66.3	88.0	67.4	53.5	19.5	-8.8	-15.3	-23.6
	3.98	20	0.60 (1.03)	0.69 (0.86)	0.58 (0.93)	0.283 (0.422)	0.284 (0.419)	0.276 (0.407)	73.5	78.3	88.0	48.0	30.6	16.7	-7.0	-11.5	-15.9
3EZM (S22)	1.74	10	0.07 (0.21)	0.08 (0.23)	0.08 (0.25)	0.266 (0.306)	0.267 (0.310)	0.267 (0.310)	99.0	98.0	99.0	2.6	2.0	1.3	-12.6	-15.9	-16.1
	1.85	10	0.07 (0.21)	0.08 (0.22)	0.08 (0.23)	0.260 (0.291)	0.260 (0.291)	0.262 (0.291)	98.0	98.0	98.0	2.0	0.7	0.7	-13.2	-15.5	-15.9

PDB ID (ref)	Synth. Res. (Å)	w_{HINT}	RMSD vs. PDB (Å): Cα (all atom)			R (R_{free})			Ramachandran score			Clashscore			E_{HINT} (kcal mol^{-1})		
			0	0+e	0+H	0	0+e	0+H	0	0+e	0+H	0	0+e	0+H	0	0+e	0+H
3EZM (cont.)	2.01	20	0.09 (0.23)	0.09 (0.25)	0.10 (0.24)	0.257 (0.299)	0.256 (0.300)	0.260 (0.300)	98.0	98.0	97.0	2.6	2.0	2.0	-12.7	-15.2	-15.7
	2.19	10	0.10 (0.24)	0.11 (0.25)	0.11 (0.25)	0.252 (0.283)	0.253 (0.280)	0.252 (0.279)	99.0	98.0	99.0	4.0	4.0	2.6	-12.7	-14.9	-14.3
	2.39	30	0.13 (0.30)	0.13 (0.36)	0.15 (0.30)	0.237 (0.281)	0.238 (0.279)	0.248 (0.277)	99.0	99.0	98.0	3.3	3.3	3.3	-12.2	-15.4	-18.4
	2.58	10	0.17 (0.32)	0.18 (0.33)	0.18 (0.32)	0.228 (0.284)	0.230 (0.289)	0.232 (0.282)	98.0	98.0	98.0	8.0	7.3	6.0	-12.7	-15.4	-14.7
	2.69	50	0.18 (0.33)	0.18 (0.42)	0.22 (0.33)	0.226 (0.304)	0.223 (0.294)	0.240 (0.294)	98.0	97.0	93.9	6.6	8.6	4.6	-13.2	-15.9	-21.9
	2.83	40	0.19 (0.35)	0.20 (0.43)	0.22 (0.36)	0.211 (0.285)	0.212 (0.295)	0.228 (0.293)	96.0	96.0	92.9	7.3	6.0	3.3	-11.5	-14.6	-20.0
	2.96	20	0.21 (0.38)	0.22 (0.40)	0.22 (0.38)	0.217 (0.316)	0.217 (0.312)	0.222 (0.318)	96.0	98.0	96.0	7.3	6.6	4.6	-12.3	-15.8	-15.3
	3.07	40	0.24 (0.46)	0.27 (0.49)	0.30 (0.44)	0.201 (0.332)	0.202 (0.312)	0.213 (0.295)	96.0	94.9	91.9	9.9	11.3	2.0	-9.5	-12.7	-20.7
	3.36	10	0.30 (0.56)	0.35 (0.55)	0.32 (0.52)	0.194 (0.289)	0.191 (0.287)	0.200 (0.290)	89.9	92.9	91.9	23.9	20.5	10.6	-6.8	-11.2	-11.9
	3.65	30	0.34 (0.65)	0.38 (0.68)	0.39 (0.63)	0.195 (0.340)	0.194 (0.321)	0.194 (0.331)	84.8	87.9	86.9	21.2	17.2	11.9	-3.4	-10.8	-18.0
	3.92	10	0.42 (0.76)	0.46 (0.74)	0.44 (0.74)	0.208 (0.321)	0.209 (0.305)	0.209 (0.312)	89.9	91.9	89.9	27.8	32.5	15.9	-3.8	-5.6	-12.7
1TP6 (S23)	1.66	10	0.09 (0.20)	0.08 (0.17)	0.08 (0.19)	0.242 (0.309)	0.242 (0.325)	0.243 (0.324)	100.0	100.0	100.0	9.2	7.2	3.1	-29.6	-38.9	-33.8
	1.77	20	0.10 (0.18)	0.09 (0.25)	0.10 (0.21)	0.242 (0.272)	0.245 (0.281)	0.251 (0.274)	99.2	99.2	98.4	5.7	6.7	3.6	-28.0	-37.0	-38.6
	1.90	30	0.08 (0.17)	0.07 (0.25)	0.09 (0.16)	0.231 (0.318)	0.233 (0.315)	0.239 (0.311)	100.0	100.0	99.2	6.7	5.7	4.1	-29.4	-37.4	-39.0
	2.02	10	0.09 (0.18)	0.08 (0.18)	0.09 (0.17)	0.226 (0.302)	0.228 (0.297)	0.228 (0.315)	100.0	100.0	100.0	7.7	7.2	3.6	-30.4	-38.5	-34.8
	2.15	20	0.11 (0.21)	0.10 (0.32)	0.10 (0.23)	0.225 (0.297)	0.230 (0.306)	0.233 (0.305)	99.2	99.2	99.2	6.2	8.2	3.6	-30.1	-36.5	-38.1
	2.31	40	0.12 (0.19)	0.11 (0.35)	0.15 (0.20)	0.216 (0.310)	0.218 (0.298)	0.231 (0.310)	99.2	98.4	98.4	7.7	7.2	2.6	-27.7	-37.0	-41.1
	2.47	20	0.15 (0.27)	0.15 (0.38)	0.18 (0.27)	0.212 (0.311)	0.217 (0.316)	0.222 (0.299)	97.6	96.8	96.0	10.3	8.7	5.7	-28.3	-36.8	-36.8
	2.62	30	0.18 (0.29)	0.19 (0.30)	0.20 (0.28)	0.214 (0.373)	0.214 (0.317)	0.220 (0.323)	96.8	96.0	95.2	9.8	10.8	4.6	-26.8	-35.0	-38.9
	2.74	30	0.21 (0.38)	0.23 (0.38)	0.21 (0.32)	0.229 (0.397)	0.225 (0.406)	0.222 (0.385)	97.6	97.6	98.4	16.4	15.4	2.6	-26.4	-38.4	-42.0
	2.87	40	0.22 (0.37)	0.22 (0.43)	0.24 (0.36)	0.197 (0.307)	0.199 (0.329)	0.206 (0.290)	94.4	95.2	93.5	15.4	17.0	3.1	-25.5	-38.0	-43.0
1V8E (S24)	1.64	10	0.17 (0.22)	0.15 (0.23)	0.16 (0.25)	0.257 (0.274)	0.257 (0.273)	0.258 (0.274)	97.7	97.7	98.1	6.0	6.9	3.9	-58.1	-69.8	-61.6
	1.81	10	0.16 (0.23)	0.15 (0.24)	0.15 (0.26)	0.248 (0.277)	0.249 (0.281)	0.249 (0.278)	97.7	98.1	97.7	6.3	8.3	3.9	-57.1	-68.8	-61.1
	1.99	10	0.16 (0.27)	0.15 (0.29)	0.16 (0.27)	0.243 (0.261)	0.244 (0.262)	0.243 (0.261)	97.2	97.2	97.2	5.1	6.3	4.8	-57.5	-69.5	-60.9
	2.20	20	0.17 (0.25)	0.15 (0.27)	0.17 (0.28)	0.234 (0.261)	0.237 (0.261)	0.237 (0.261)	96.7	96.7	96.7	6.9	8.9	3.0	-53.6	-68.1	-61.8
	2.36	20	0.18 (0.30)	0.18 (0.30)	0.18 (0.32)	0.224 (0.261)	0.225 (0.262)	0.226 (0.262)	96.7	97.7	96.7	5.4	6.0	4.5	-54.1	-67.2	-62.1
	2.52	30	0.21 (0.33)	0.20 (0.31)	0.20 (0.33)	0.216 (0.263)	0.218 (0.259)	0.221 (0.265)	96.7	97.2	97.2	10.1	10.1	4.2	-52.2	-65.7	-63.6
	2.66	10	0.23 (0.34)	0.20 (0.34)	0.22 (0.37)	0.209 (0.266)	0.210 (0.265)	0.210 (0.263)	96.3	97.7	95.8	8.6	9.2	7.5	-53.6	-66.8	-57.7
	2.82	10	0.22 (0.38)	0.22 (0.37)	0.20 (0.38)	0.202 (0.266)	0.203 (0.262)	0.203 (0.260)	94.0	94.4	95.8	12.5	14.3	7.8	-50.4	-65.7	-55.8
	3.01	20	0.25 (0.43)	0.25 (0.43)	0.25 (0.42)	0.195 (0.281)	0.194 (0.277)	0.197 (0.272)	93.5	94.4	94.9	21.8	21.2	11.3	-47.9	-64.0	-61.4
	3.13	30	0.35 (0.55)	0.35 (0.47)	0.30 (0.54)	0.218 (0.322)	0.213 (0.310)	0.221 (0.312)	92.6	94.9	94.9	20.9	19.4	8.3	-44.2	-62.0	-62.3
	3.46	30	0.43 (0.63)	0.42 (0.60)	0.37 (0.63)	0.211 (0.337)	0.216 (0.345)	0.216 (0.313)	91.6	89.3	89.8	31.0	28.3	9.5	-36.6	-56.5	-64.2
	3.78	40	0.45 (0.80)	0.49 (0.73)	0.46 (0.76)	0.230 (0.344)	0.230 (0.343)	0.232 (0.324)	79.1	81.4	89.3	59.0	56.9	15.8	-22.1	-41.7	-63.0
	4.11	10	0.52 (0.82)	0.53 (0.78)	0.52 (0.81)	0.205 (0.355)	0.204 (0.342)	0.198 (0.348)	75.8	79.1	79.1	44.7	53.1	29.2	-19.4	-43.6	-41.1
	4.39	60	0.68 (1.01)	0.69 (0.85)	0.56 (0.98)	0.272 (0.402)	0.293 (0.424)	0.276 (0.387)	69.8	73.5	81.9	75.4	62.0	12.2	-7.6	-35.1	-73.6
1OI7 (S25)	1.49	20	0.08 (0.21)	0.10 (0.24)	0.08 (0.24)	0.243 (0.263)	0.237 (0.256)	0.236 (0.254)	97.0	97.4	96.6	5.2	2.7	1.5	-66.5	-77.8	-77.9
	1.71	10	0.14 (0.22)	0.10 (0.22)	0.10 (0.40)	0.238 (0.259)	0.233 (0.256)	0.239 (0.257)	97.4	97.0	97.4	6.5	4.0	5.2	-65.5	-80.5	-69.8
	1.85	20	0.10 (0.22)	0.11 (0.26)	0.11 (0.24)	0.234 (0.253)	0.229 (0.252)	0.229 (0.251)	97.4	97.7	96.6	6.7	4.7	2.5	-64.2	-77.3	-77.6
	2.02	30	0.14 (0.26)	0.11 (0.35)	0.11 (0.29)	0.223 (0.262)	0.220 (0.261)	0.226 (0.262)	96.6	96.6	96.6	5.0	5.2	1.7	-65.9	-78.2	-79.0
	2.18	10	0.13 (0.27)	0.12 (0.26)	0.13 (0.29)	0.216 (0.248)	0.215 (0.247)	0.218 (0.245)	96.2	95.9	96.2	4.7	6.0	5.2	-69.3	-78.6	-70.4
	2.37	30	0.14 (0.28)	0.14 (0.28)	0.14 (0.28)	0.218 (0.252)	0.219 (0.254)	0.218 (0.248)	96.6	97.0	96.6	9.0	10.0	5.5	-60.2	-66.5	-70.7
	2.54	10	0.15 (0.28)	0.16 (0.28)	0.15 (0.29)	0.209 (0.263)	0.205 (0.261)	0.206 (0.261)	97.0	95.9	97.0	11.0	7.2	7.5	-62.4	-74.2	-69.0
	2.69	50	0.17 (0.30)	0.17 (0.31)	0.18 (0.32)	0.206 (0.258)	0.201 (0.262)	0.210 (0.255)	96.6	95.5	97.0	10.2	8.7	3.5	-59.6	-71.1	-74.1
	2.84	50	0.20 (0.39)	0.21 (0.38)	0.21 (0.35)	0.192 (0.265)	0.200 (0.262)	0.200 (0.248)	93.6	94.4	94.7	14.7	16.9	7.5	-54.9	-60.1	-76.6
	2.97	60	0.22 (0.41)	0.22 (0.38)	0.22 (0.39)	0.189 (0.288)	0.190 (0.289)	0.198 (0.283)	94.4	95.1	95.9	15.4	16.9	7.0	-54.4	-64.0	-78.0
	3.28	50	0.33 (0.53)	0.29 (0.47)	0.27 (0.58)	0.199 (0.287)	0.200 (0.302)	0.198 (0.269)	90.6	89.1	91.7	29.9	26.7	9.2	-41.2	-55.3	-75.5
	3.57	60	0.36 (0.60)	0.37 (0.53)	0.32 (0.58)	0.194 (0.314)	0.195 (0.309)	0.194 (0.290)	86.8	87.2	91.0	45.9	46.4	9.7	-32.2	-37.7	-80.2
	3.83	60	0.45 (0.72)	0.47 (0.64)	0.40 (0.70)	0.224 (0.343)	0.219 (0.354)	0.211 (0.309)	81.2	83.8	91.4	45.6	46.9	15.9	-23.1	-34.6	-74.7
	4.07	60	0.62 (0.89)	0.62 (0.74)	0.50 (0.92)	0.223 (0.361)	0.224 (0.352)	0.217 (0.334)	68.4	68.0	79.7	84.2	101.9	21.7	5.0	10.4	-70.0

References (for Tables S1 and S2)

S1. McCleverty CJ, Liddington RC (2003) Engineered allosteric mutants of the integrin alphaMbeta2 I domain, structural and functional studies. Biochem J 372: 121-127.

S2. Dock-Bregeon A-C, Rees B, Torres-Larios A, Bey G, Caillet J, et al. (2004) Achieving error-free translation; the mechanism of proofreading of threonyl-tRNA synthetase at atomic resolution. Mol Cell 16: 375-386.

S3. Li W-F, Yu J, Ma X-X, Teng Y-B, Luo M, et al. (2010) Structural basis for the different activities of yeast Grx1 and Grx2. Biochim Biophys Acta 1804: 1542-1547.

S4. Osawa T, Matsubara Y, Muramatsu T, Kimura M, Kakuta Y (2005) Crystal structure of the alginate (poly α-l-guluronate) lyase from Corynebacterium sp. at 1.2 Å resolution. J Mol Biol 345: 1111-1118.

S5. Park EY, Kwon O-B, Jeong B-C, Yi J-S, Lee CS, et al. (2010) Crystal structure of PRY-SPRY domain of human TRIM72. Proteins 78: 790-795.

S6. Koropatkin NM, Koppenaal DW, Pakrasi HB, Smith TJ (2007) The structure of a cyanobacterial bicarbonate transport protein, CmpA. J Biol Chem 282: 2606-2614.

S7. Boisvert DC, Kim SH A structural approach to gene function and structure quality for Pyrococcus horikoshii Fibrillarin. To Be Published.

S8. Karthikeyan S, Leung T, Birrane G, Webster G, Ladias JA (2001) Crystal structure of the PDZ1 domain of human Na^+/H^+ exchanger regulatory factor provides insights into the mechanism of carboxyl-terminal leucine recognition by class I PDZ domains. J Mol Biol 308: 963-973.

S9. Vorobiev SM, Kuzin A, Abashidze M, Forouhar F, Xio R, et al. Crystal structure of hypothetical protein Atu071 from Agobacterium tumefaciens. Northeast Structural Genomics Consortium Target AtR8. To Be Published.

S10. Chen L, Chen L-R, Zhou XE, Wang Y, Kahsai MA, et al. (2004) The hyperthermophile protein Sso10a is a dimer of winged helix DNA-binding domains linked by an antiparallel coiled coil rod. J Mol Biol 341: 73-91.

S11. Olsen JG, Flensburg C, Olsen O, Bricogne G, Henriksen A (2004) Solving the structure of the bubble protein using the anomalous sulfur signal from single-crystal in-house Cu Kα diffraction data only. Acta Crystallogr D 60: 250-255.

S12. Fermani S, Falini G, Ripamonti A, Polito L, Stirpe F, et al. (2005) The 1.4 Å structure of dianthin 30 indicates a role of surface potential at the active site of type 1 ribosome inactivating proteins. J Struct Biol 149: 204-212.

S13. Zhang R, Skarina T, Savchenko A, Edwards A, Joachimiak A 1.5 Å crystal structure of a hypothetical protein APE0754 from Aeropyrum pernix. To Be Published.

S14. Aibara S, Suzuki A, Kidera A, Shibata K, Yamane T, et al. The crystal structure of the orthorhombic form of hen egg white lysozyme at 1.5 angstroms resolution. To Be Published.

S15. Meng J, Vardar D, Wang Y, Guo H-C, Head JF, et al. (2005) High-resolution crystal structures of villin headpiece and mutants with reduced F-actin binding activity. Biochemistry 44: 11963-11973.

S16. Gould AD, Shilton BH (2010) Studies of the maltose transport system reveal a mechanism for coupling ATP hydrolysis to substrate translocation without direct recognition of substrate. J Biol Chem 285:11290-11296.

S17. Shao H, Dixon RA, Wang X (2007) Crystal structure of vestitone reductase from alfalfa (Medicago sativa L.). J Mol Biol 369:265-276.

S18. Fedorov AA, Domagalski M, Fedorov EV, Toro R, Sauder JM, et al. Crystal structure of glutamine amido transferase from Methylobacillus Flagellatus. To Be Published.

S19. Li Y, Fanning AS, Anderson JM, Lavie A (2005) Structure of the conserved cytoplasmic C-terminal domain of occludin, identification of the ZO-1 binding surface. J Mol Biol 352:151-164.

S20. Chiba-Kamoshida K, Matsui T, Chatake T, Ohhara T, Ostermann A, et al. Site-specific softening of peptide bonds by localized deuterium observed by neutron crystallography of human lysozyme hydrogen. To Be Published.

S21. Da G, Lenkart J, Zhao K, Shiekhattar R, Cairns BR, et al. (2006) Structure and function of the SWIRM domain, a conserved protein module found in chromatin regulatory complexes. Proc Natl Acad Sci U S A 103: 2057-2062.

S22. Yang F, Bewley CA, Louis JM, Gustafson KR, Boyd MR, et al. (1999) Crystal structure of cyanovirin-N, a potent HIV-inactivating protein, shows unexpected domain swapping. J Mol Biol 288: 403-412.

S23. Zhang R, Xu LX, Savchenko A, Edwards A, Joachimiak A 1.5 Å crystal structure of a hypothetical protein PA1314 from Pseudomonas aeruginosa. To Be Published.

S24. Ishijima J, Ida K, Yutani K, Miyano M Crystal structure of glycerophosphoryl diester phosphodiesterase from Thermus thermophilus HB8. To Be Published.
S25. Takahashi H, Tokunaga Y, Kuroishi C, Babayeba N, Kuramitsu S, et al. The Crystal Structure of Succinyl-Coa Synthetase from Thermus Thermophilus. To Be Published.
