# Supplementary material for: Applying an Empirical Hydropathic Forcefield in Refinement May Improve Low-Resolution Protein X-Ray Crystal Structures
Source: PLoS One. 2011 Jan 5;6(1):e15920. doi: 10.1371/journal.pone.0015920 (PMC3016398; doi:10.1371/journal.pone.0015920)
Supplement: Table S2 — Crystallographic statistics for re-refined high-resolution structures. Refinement protocols: 0 = native CNS v. 1.1; 0+e = CNS+electrostatics; 0+H = CNS+HINT. See Table S1 for references. (DOC) [file pone.0015920.s005.doc]

PDB ID (ref)	Synth. Res. (Å)	No. reflect.	completeness	RMS bond (Å)			RMS angle (deg.)			PDB ID (ref)	Synth. Res. (Å)	No. reflect.	completeness	RMS bond (Å)			RMS angle (deg.)		
				0	0+e	0+H	0	0+e	0+H					0	0+e	0+H	0	0+e	0+H
1NA5 (S1)	1.73	20965	96.9	0.0046	0.0050	0.0054	0.6909	0.7203	0.7223	3KB5 (S5)	1.80	16415	97.5	0.0049	0.0051	0.0056	0.7099	0.7665	0.7346
	1.89	16228	97.2	0.0050	0.0051	0.0056	0.6982	0.7271	0.7310		1.91	13846	97.9	0.0052	0.0057	0.0058	0.7231	0.7896	0.7515
	2.07	12422	97.1	0.0054	0.0056	0.0072	0.7075	0.7311	0.7873		2.02	11754	98.2	0.0059	0.0068	0.0086	0.7376	0.7906	0.8720
	2.26	9569	96.8	0.0056	0.0059	0.0062	0.7101	0.7543	0.7417		2.13	10032	98.2	0.0059	0.0068	0.0065	0.7577	0.7441	0.7712
	2.48	7289	96.6	0.006	0.006	0.0063	0.7190	0.7433	0.7409		2.25	8537	98.1	0.0061	0.0068	0.0071	0.7594	0.7487	0.7891
	2.65	5997	96.4	0.0064	0.0064	0.0065	0.7417	0.7704	0.7623		2.39	7115	97.9	0.0068	0.0072	0.0077	0.7587	0.7156	0.7860
	3.00	4151	96.0	0.0067	0.0067	0.0073	0.7351	0.7564	0.7915		2.51	6159	97.7	0.0070	0.0079	0.0089	0.7620	0.8069	0.8938
	3.16	3555	95.6	0.0072	0.0075	0.008	0.7367	0.7875	0.8035		2.62	5420	97.6	0.0074	0.0073	0.0072	0.7632	0.8041	0.7672
	3.33	3057	95.3	0.0073	0.0071	0.0079	0.7412	0.7533	0.7994		2.87	4118	97.0	0.0070	0.0078	0.0076	0.7549	0.8113	0.7965
	3.72	2193	94.3	0.0073	0.0071	0.0079	0.7191	0.7476	0.7845		3.13	3176	96.4	0.0076	0.0088	0.0100	0.7985	0.8229	0.9147
	4.09	1653	93.6	0.0076	0.0076	0.0083	0.7431	0.7471	0.8066		3.37	2534	95.8	0.0085	0.0091	0.0091	0.7911	0.8636	0.8837
	4.40	1327	93.3	0.0078	0.0075	0.0085	0.7782	0.7384	0.8085		3.59	2090	95.3	0.0080	0.0084	0.0077	0.7859	0.7515	0.7796
	4.64	1128	92.6	0.0076	0.0077	0.0088	0.7811	0.8263	0.8461		3.79	1771	94.9	0.0077	0.0094	0.0100	0.7624	0.9282	0.9321
1TJE (S2)	1.73	22033	88.8	0.0047	0.0049	0.0052	0.6469	0.6721	0.6693		4.00	1507	94.4	0.0085	0.0094	0.0095	0.8327	0.8783	0.9102
	1.95	16121	92.2	0.0051	0.0053	0.0054	0.6574	0.6847	0.6685	2I49 (S6)	1.57	66060	99.9	0.0047	0.0048	0.0053	0.7423	0.7586	0.7669
	2.19	11949	96.1	0.0059	0.0061	0.0062	0.6758	0.6990	0.6877		1.76	46886	100.0	0.0048	0.0049	0.0053	0.7265	0.7422	0.7459
	2.45	8852	98.8	0.0063	0.0063	0.0067	0.6943	0.7193	0.7206		1.92	36063	100.0	0.0052	0.0053	0.0056	0.7175	0.7357	0.7363
	2.66	7019	99.5	0.0061	0.0063	0.0064	0.6902	0.7239	0.7080		2.07	28797	99.9	0.0058	0.0057	0.0072	0.7083	0.7267	0.7764
	2.84	5805	99.6	0.0067	0.0068	0.0072	0.6937	0.7188	0.7280		2.22	23323	99.9	0.0060	0.0060	0.0078	0.7277	0.7383	0.8089
	3.00	4962	99.7	0.0066	0.0068	0.0072	0.7062	0.7345	0.7438		2.37	19175	99.9	0.0060	0.0060	0.0068	0.7172	0.7381	0.7577
	3.17	4225	99.7	0.0064	0.0064	0.0070	0.6938	0.7166	0.7220		2.52	15963	100.0	0.0065	0.0064	0.0070	0.7373	0.7537	0.7577
	3.31	3721	99.7	0.0069	0.0068	0.0082	0.7420	0.7618	0.8127		2.66	13565	100.0	0.0063	0.0063	0.0070	0.7367	0.7530	0.7741
	3.48	3218	99.7	0.0073	0.0072	0.0075	0.7525	0.7606	0.7698		2.79	11746	99.9	0.0068	0.0067	0.0091	0.7406	0.7560	0.8696
	3.87	2375	99.5	0.0072	0.0075	0.0093	0.7660	0.7767	0.8700		2.92	10246	99.9	0.0066	0.0065	0.0069	0.7438	0.7561	0.7636
	4.23	1837	99.4	0.0087	0.0071	0.0107	0.8320	0.7317	0.9345		3.21	7703	100.0	0.0069	0.0066	0.0088	0.7410	0.7387	0.8647
	4.56	1477	99.3	0.0067	0.0074	0.0092	0.7231	0.7544	0.8571		3.48	6071	99.9	0.0070	0.0069	0.0088	0.7462	0.7514	0.8495
	4.88	1213	99.2	0.0073	0.0081	0.0096	0.7681	0.8000	0.8783		3.73	4896	100.0	0.0075	0.0071	0.0090	0.7383	0.7392	0.8556
3CTG (S3)	1.71	14096	97.5	0.0045	0.0042	0.0047	0.7060	0.7401	0.7239		3.95	4134	99.9	0.0077	0.0076	0.0091	0.7428	0.7414	0.8484
	1.93	9883	98.2	0.0053	0.0059	0.0056	0.6907	0.6625	0.7094	1G8A (S7)	1.62	25826	99.7	0.0046	0.0049	0.0051	0.7357	0.7512	0.7495
	2.20	6712	98.6	0.0062	0.0064	0.0065	0.7247	0.7090	0.7406		1.96	14685	99.7	0.0055	0.0056	0.0075	0.7408	0.7689	0.8170
	2.46	4807	98.7	0.0070	0.0071	0.0072	0.7425	0.7280	0.7521		2.34	8663	99.6	0.0063	0.0065	0.0082	0.7302	0.7711	0.8199
	2.69	3690	98.6	0.0069	0.0074	0.0070	0.7667	0.7876	0.7599		2.71	5598	99.4	0.0071	0.0070	0.0086	0.7590	0.7640	0.8472
	2.93	2857	98.5	0.0072	0.0087	0.0089	0.7339	0.7798	0.8642		3.04	3994	99.3	0.0076	0.0074	0.0092	0.7829	0.7898	0.9019
	3.14	2313	98.2	0.0073	0.0082	0.0077	0.7420	0.7495	0.7682		3.34	3024	99.1	0.0080	0.0074	0.0094	0.7549	0.7501	0.8689
	3.38	1853	97.9	0.0079	0.0093	0.0092	0.7485	0.7347	0.8622		3.59	2429	98.9	0.0076	0.0074	0.0081	0.7475	0.7538	0.8009
	3.59	1544	97.5	0.0076	0.0082	0.0085	0.7524	0.7504	0.9117		3.88	1925	98.6	0.0092	0.0080	0.0096	0.8197	0.8042	0.8529
1UAI (S4)	1.48	31977	99.5	0.0043	0.0048	0.0050	0.8082	0.8342	0.8353		4.08	1660	98.4	0.0094	0.0089	0.0093	0.9586	0.9165	0.8434
	1.58	26306	99.5	0.0044	0.0049	0.0051	0.8046	0.8346	0.8362		4.29	1430	98.3	0.0083	0.0085	0.0093	0.7986	0.8553	0.8798
	1.85	16426	99.3	0.0050	0.0053	0.0055	0.7880	0.8162	0.8212		4.72	1082	97.9	0.0073	0.0084	0.0091	0.7108	0.7819	0.8373
	2.17	10200	99.0	0.0058	0.0061	0.0062	0.7972	0.8263	0.8194	1G9O (S8)	1.67	10841	99.5	0.0044	0.0047	0.0049	0.6944	0.7081	0.7131
	2.52	6525	98.5	0.0067	0.0067	0.0088	0.7911	0.8196	0.9354		1.92	7214	99.6	0.0049	0.0052	0.0054	0.6822	0.7097	0.7016
	2.65	5602	98.3	0.0063	0.0065	0.0086	0.7650	0.8016	0.9192		2.19	4910	99.5	0.0053	0.0055	0.0056	0.6877	0.7059	0.7005
	2.78	4847	98.1	0.0068	0.0070	0.0087	0.7924	0.8122	0.9088		2.44	3573	99.3	0.0062	0.0063	0.0066	0.7343	0.7494	0.7460
	2.92	4187	97.9	0.0072	0.0072	0.0076	0.7953	0.8200	0.8128		2.64	2839	99.1	0.0060	0.0065	0.0062	0.6996	0.7283	0.7018
	3.06	3644	97.6	0.0067	0.0067	0.0085	0.7559	0.7800	0.9099		2.87	2218	98.8	0.0064	0.0065	0.0070	0.7540	0.7560	0.7652
	3.20	3176	97.3	0.0071	0.0071	0.0091	0.7913	0.8123	0.9244		3.10	1769	98.6	0.0066	0.0066	0.0078	0.7099	0.7306	0.7842
	3.35	2759	97.0	0.0073	0.0075	0.0088	0.7926	0.8080	0.9321		3.20	1617	98.4	0.0069	0.0066	0.0075	0.7749	0.7708	0.7796
	3.52	2396	96.8	0.0076	0.0075	0.0076	0.8031	0.8160	0.8279		3.33	1444	98.3	0.0071	0.0070	0.0076	0.7629	0.7602	0.7843
	3.65	2134	96.6	0.0084	0.0082	0.0077	0.8391	0.8514	0.8296		3.48	1262	98.1	0.0068	0.0076	0.0079	0.7472	0.7544	0.8129
	3.77	1943	96.4	0.0093	0.0083	0.0100	0.8899	0.8649	0.9507		3.94	873	97.3	0.0091	0.0081	0.0091	0.8150	0.8248	0.8534
	3.92	1731	96.2	0.0067	0.0070	0.0100	0.7285	0.7689	0.9544	1ZHV	1.69	14082	49.5	0.0043	0.0047	0.0076	0.7387	0.7798	0.8743

PDB ID (ref)	Synth. Res. (Å)	No. reflect.	completeness	RMS bond (Å)			RMS angle (deg.)			PDB ID (ref)	Synth. Res. (Å)	No. reflect.	completeness	RMS bond (Å)			RMS angle (deg.)		
				0	0+e	0+H	0	0+e	0+H					0	0+e	0+H	0	0+e	0+H
1ZHV (S9) (cont.)	1.85	10777	49.4	0.0051	0.0053	0.0057	0.7452	0.7754	0.7740	1TUA (cont.)	2.46	7526	98.6	0.0069	0.0068	0.0072	0.6549	0.6928	0.6828
	2.03	8205	49.4	0.0053	0.0057	0.0067	0.7671	0.7978	0.8187		2.62	6238	98.5	0.0072	0.0070	0.0080	0.6556	0.6584	0.6885
	2.27	5909	49.4	0.0061	0.0061	0.0087	0.7699	0.8055	0.8948		2.75	5423	98.5	0.0070	0.0071	0.0092	0.6780	0.7013	0.8376
	2.49	4502	49.3	0.0069	0.0072	0.0090	0.7644	0.8005	0.8867		2.88	4712	98.3	0.0069	0.0069	0.0071	0.6453	0.6737	0.6633
	2.69	3601	49.3	0.0067	0.0063	0.0054	0.7655	0.7591	0.6654		3.00	4174	98.2	0.0067	0.0064	0.0071	0.6484	0.6574	0.6724
	2.89	2885	48.9	0.0063	0.0067	0.0077	0.6762	0.7274	0.7833		3.26	3261	98.0	0.0077	0.0074	0.0075	0.6809	0.7368	0.7064
	3.06	2449	48.9	0.0068	0.0067	0.0093	0.7264	0.7487	0.8776		3.52	2599	97.7	0.0084	0.0081	0.0094	0.8003	0.7942	0.8135
	3.25	2045	48.7	0.0080	0.0082	0.0091	0.8229	0.8359	0.8523		3.79	2093	97.4	0.0079	0.0072	0.0091	0.7780	0.6964	0.8007
	3.50	1636	48.3	0.0093	0.0072	0.0064	0.9433	0.7256	0.6425	1VDQ (S14)	1.68	13464	91.1	0.0045	0.0047	0.0050	0.7550	0.7589	0.7714
	3.76	1351	49.0	0.0086	0.0080	0.0085	0.8226	0.7613	0.8871		1.85	10182	91.5	0.0051	0.0052	0.0055	0.7423	0.7565	0.7643
1R7J (S10)	1.58	17597	97.2	0.0038	0.0041	0.0043	0.5967	0.6244	0.6144		2.07	7325	91.3	0.0052	0.0051	0.0070	0.7420	0.7430	0.8197
	1.71	13994	97.6	0.0042	0.0045	0.0046	0.6163	0.6482	0.6285		2.33	5126	90.2	0.0060	0.0058	0.0062	0.7597	0.7561	0.7704
	1.93	9892	98.2	0.0051	0.0052	0.0059	0.6088	0.6417	0.6560		2.59	3700	88.5	0.0060	0.0059	0.0068	0.7532	0.7555	0.7744
	2.04	8432	98.5	0.0055	0.0056	0.0059	0.6142	0.6372	0.6367		2.79	2935	87.4	0.0058	0.0058	0.0063	0.7171	0.7194	0.7397
	2.16	7134	98.4	0.0054	0.0055	0.0061	0.6325	0.6581	0.6519		3.02	2297	85.8	0.0060	0.0058	0.0067	0.7131	0.7018	0.7613
	2.31	5870	98.6	0.0063	0.0064	0.0079	0.6217	0.6474	0.7014		3.23	1868	84.6	0.0059	0.0063	0.0064	0.6887	0.7314	0.7210
	2.47	4829	98.3	0.0060	0.0060	0.0064	0.6607	0.6663	0.6756		3.39	1598	83.4	0.0064	0.0060	0.0085	0.7147	0.7274	0.8606
	2.57	4294	98.2	0.0062	0.0062	0.0069	0.6536	0.6662	0.6841		3.59	1340	82.3	0.0058	0.0060	0.0064	0.6748	0.7050	0.7040
	2.70	3714	98.1	0.0067	0.0066	0.0073	0.6465	0.6755	0.6853		4.01	948	80.2	0.0071	0.0064	0.0080	0.6785	0.7017	0.7856
	2.83	3236	97.9	0.0065	0.0063	0.0122	0.6459	0.6760	0.9413	1YU5 (S15)	1.68	7521	99.3	0.0045	0.0047	0.0049	0.6891	0.7031	0.7082
	3.24	2159	96.9	0.0083	0.0086	0.0095	0.6840	0.7136	0.7882		1.93	4998	98.9	0.0056	0.0057	0.0065	0.6950	0.6992	0.7345
	3.59	1600	96.0	0.0074	0.0075	0.0079	0.6315	0.6456	0.6616		2.14	3692	98.5	0.0061	0.0060	0.0067	0.6923	0.7061	0.7381
	3.96	1195	94.9	0.0064	0.0059	0.0094	0.6304	0.6005	0.8017		2.40	2626	97.9	0.0069	0.0066	0.0076	0.7333	0.7378	0.7136
	4.20	998	94.2	0.0088	0.0073	0.0109	0.7347	0.6805	0.8586		2.61	2053	97.4	0.0071	0.0068	0.0081	0.7620	0.7746	0.8170
1UOY (S11)	1.68	8060	100.0	0.0046	0.0048	0.0054	0.7830	0.7776	0.8066		2.74	1777	97.0	0.0070	0.0066	0.0071	0.8007	0.7778	0.7846
	2.13	4018	100.0	0.0056	0.0056	0.0066	0.7749	0.7677	0.8136		2.80	1672	96.8	0.0066	0.0071	0.0075	0.7130	0.7426	0.7676
	2.45	2658	100.0	0.0065	0.0067	0.0088	0.8065	0.8110	0.8602		2.99	1374	96.2	0.0072	0.0071	0.0078	0.7887	0.7722	0.8188
	2.72	1973	99.9	0.0067	0.0066	0.0076	0.7451	0.7822	0.8245		3.60	782	93.4	0.0080	0.0083	0.0096	0.7566	0.7918	0.9379
	3.11	1325	99.9	0.0078	0.0077	0.0083	0.7964	0.7944	0.8073		3.68	741	93.1	0.0085	0.0070	0.0099	0.7916	0.7552	0.8680
	3.29	1127	99.9	0.0068	0.0075	0.0306	0.7442	0.8149	3.0260		3.75	696	92.9	0.0082	0.0079	0.0088	0.7551	0.7858	0.8736
	3.58	877	99.9	0.0083	0.0076	0.0095	0.8294	0.8376	0.8936		4.08	541	92.3	0.0080	0.0065	0.0088	0.7614	0.7102	0.8891
	3.78	756	99.9	0.0075	0.0065	0.0093	0.7378	0.7466	0.9142	3KJT (S16)	1.71	33912	97.9	0.0048	0.0049	0.0053	0.6669	0.6876	0.6859
	4.01	627	99.8	0.0067	0.0069	0.0086	0.7154	0.7479	0.8880		1.87	26061	98.2	0.0053	0.0053	0.0057	0.6707	0.6929	0.6928
1RL0 (S12)	1.70	25642	96.4	0.0081	0.0080	0.0085	0.7551	0.7693	0.7711		1.99	21681	98.4	0.0057	0.0057	0.0066	0.6797	0.7026	0.7209
	2.12	13587	98.6	0.0080	0.0078	0.0083	0.7616	0.7768	0.7760		2.11	18226	98.4	0.0061	0.0062	0.0083	0.6979	0.7185	0.8144
	2.33	10273	98.8	0.0082	0.0078	0.0086	0.7695	0.7782	0.7919		2.25	15031	98.4	0.0067	0.0066	0.0070	0.7090	0.7281	0.7302
	2.42	9165	98.8	0.0081	0.0080	0.0099	0.7767	0.7830	0.8434		2.40	12405	98.4	0.0068	0.0067	0.0078	0.7207	0.7436	0.7773
	2.58	7577	98.6	0.0082	0.0079	0.0088	0.7929	0.7974	0.8288		2.54	10483	98.3	0.0072	0.0069	0.0077	0.7298	0.7517	0.7520
	2.73	6373	98.4	0.0076	0.0074	0.0083	0.8011	0.8067	0.8330		2.64	9327	98.2	0.0074	0.0073	0.0088	0.7235	0.7482	0.8007
	2.87	5481	98.2	0.0080	0.0079	0.0087	0.7982	0.8080	0.8205		2.78	8004	98.1	0.0070	0.0069	0.0080	0.7262	0.7488	0.7928
	2.95	5063	98.0	0.0079	0.0077	0.0094	0.8082	0.8120	0.8862		2.89	7105	98.0	0.0070	0.0067	0.0081	0.7182	0.7445	0.7845
	3.06	4512	97.8	0.0082	0.0081	0.0090	0.8180	0.8201	0.8739		3.13	5591	97.7	0.0076	0.0072	0.0097	0.7458	0.7509	0.8974
	3.18	4021	97.6	0.0078	0.0075	0.0081	0.7963	0.7841	0.8280		3.28	4851	97.4	0.0076	0.0075	0.0097	0.7731	0.7770	0.8893
	3.52	2953	96.9	0.0081	0.0079	0.0093	0.7685	0.7982	0.8605		3.52	3941	97.2	0.0076	0.0073	0.0091	0.7255	0.7467	0.8565
	3.94	2099	96.3	0.0078	0.0077	0.0092	0.7952	0.7917	0.8660		3.73	3298	96.9	0.0089	0.0083	0.0099	0.8289	0.8004	0.8651
	4.14	1803	95.9	0.0081	0.0076	0.0105	0.7937	0.7950	0.9542	2P4H (S17)	1.60	50165	93.3	0.0047	0.0049	0.0050	0.7435	0.7505	0.7405
	4.31	1599	95.7	0.0079	0.0070	0.0092	0.7664	0.7368	0.8488		1.83	33638	93.0	0.0049	0.0054	0.0061	0.7335	0.7629	0.7798
1TUA (S13)	1.69	22648	97.9	0.0047	0.0050	0.0052	0.6280	0.6520	0.6427		2.07	23079	91.7	0.0067	0.0059	0.0062	0.7699	0.7439	0.7473
	1.85	17415	98.4	0.0050	0.0053	0.0061	0.6111	0.6510	0.6670		2.33	15913	89.6	0.0059	0.0062	0.0069	0.7266	0.7652	0.8024
	1.98	14276	98.5	0.0059	0.0059	0.0061	0.6362	0.6567	0.6444		2.45	13575	88.5	0.0073	0.0065	0.0078	0.7891	0.7703	0.8076
	2.13	11519	98.6	0.0061	0.0064	0.0072	0.6333	0.6546	0.6852		2.66	10431	86.6	0.0073	0.0080	0.0088	0.7721	0.8371	0.8463
	2.29	9299	98.6	0.0063	0.0062	0.0065	0.6497	0.6855	0.6685		2.87	8138	84.5	0.0067	0.0068	0.0082	0.7963	0.7932	0.8681

PDB ID (ref)	Synth. Res. (Å)	No. reflect.	completeness	RMS bond (Å)			RMS angle (deg.)		
				0	0+e	0+H	0	0+e	0+H
2P4H (cont.)	2.96	7364	83.5	0.0089	0.0076	0.0069	0.7869	0.7833	0.7557
	3.08	6449	82.2	0.0072	0.0070	0.0092	0.7920	0.8112	0.8796
	3.23	5481	80.2	0.0074	0.0093	0.0093	0.7802	0.9039	0.9080
	3.65	3613	75.6	0.0093	0.0097	0.0101	0.8554	0.7439	0.9058
	4.00	2662	72.7	0.0080	0.0075	0.0094	0.7019	0.7437	0.8163
	4.34	2050	71.0	0.0070	0.0071	0.0081	0.7407	0.7476	0.7873
	4.61	1709	70.6	0.0081	0.0071	0.0094	0.7383	0.7632	0.8553
3L83 (S18)	1.93	17472	96.9	0.0059	0.0062	0.0063	0.7011	0.7648	0.7215
	2.28	10617	97.2	0.0071	0.0069	0.0074	0.7074	0.6955	0.7344
	2.68	6504	96.8	0.0081	0.0092	0.0093	0.7518	0.7816	0.8069
	2.81	5614	96.5	0.0072	0.0076	0.0078	0.7346	0.7643	0.7709
	3.10	4158	95.8	0.0075	0.0083	0.0091	0.7369	0.7772	0.8507
	3.51	2819	94.6	0.0087	0.0087	0.0105	0.7613	0.8064	0.9121
	3.71	2447	97.2	0.0076	0.0081	0.0081	0.7102	0.7173	0.7399
	3.90	2123	98.0	0.0081	0.0090	0.0090	0.7442	0.7983	0.8327
	4.11	1819	97.8	0.0084	0.0084	0.0099	0.7693	0.8375	0.8695
	4.33	1553	97.6	0.0105	0.0105	0.0100	0.8760	0.8956	0.8381
1WPA (S19)	1.69	14640	99.1	0.0056	0.0057	0.0060	0.5511	0.5963	0.5821
	1.88	10700	98.8	0.0065	0.0062	0.0067	0.5809	0.5970	0.5921
	2.03	8528	98.5	0.0070	0.0065	0.0079	0.5932	0.5972	0.6560
	2.21	6609	98.1	0.0069	0.0064	0.0070	0.6015	0.6428	0.6144
	2.45	4882	97.5	0.0073	0.0071	0.0087	0.6346	0.6563	0.6974
	2.74	3487	96.6	0.0079	0.0070	0.0076	0.6715	0.6623	0.6838
	2.89	2975	96.0	0.0075	0.0071	0.0074	0.6755	0.6887	0.6797
	3.05	2530	95.4	0.0077	0.0076	0.0084	0.6517	0.6723	0.7130
	3.21	2161	94.7	0.0075	0.0076	0.0085	0.6933	0.7183	0.7219
	3.34	1919	94.2	0.0081	0.0078	0.0082	0.7477	0.7757	0.7531
	3.67	1433	92.5	0.0068	0.0070	0.0091	0.6210	0.6828	0.7809
	3.94	1150	91.1	0.0065	0.0066	0.0066	0.6523	0.6635	0.7304
	4.23	929	89.8	0.0079	0.0072	0.0097	0.7482	0.6621	0.8767
3FE0 (S20)	1.70	12662	94.0	0.0049	0.0050	0.0054	0.7516	0.7658	0.7695
	1.96	8382	94.4	0.0057	0.0056	0.0062	0.7613	0.7705	0.7775
	2.20	5966	94.0	0.0063	0.0061	0.0083	0.7577	0.7658	0.8180
	2.46	4264	93.0	0.0066	0.0063	0.0076	0.7653	0.7689	0.7908
	2.70	3204	91.7	0.0066	0.0062	0.0097	0.7492	0.7683	0.9003
	2.87	2655	90.8	0.0065	0.0063	0.0070	0.7571	0.7638	0.7862
	3.06	2164	89.3	0.0066	0.0066	0.0070	0.7374	0.7557	0.7570
	3.18	1919	88.4	0.0066	0.0063	0.0082	0.7450	0.7440	0.8120
	3.34	1637	87.1	0.0071	0.0066	0.0078	0.7694	0.7520	0.7751
	3.51	1397	85.5	0.0075	0.0069	0.0094	0.7597	0.7343	0.8773
	3.99	908	80.1	0.0067	0.0056	0.0090	0.7001	0.6634	0.8966
2FQ3 (S21)	1.55	14261	98.6	0.0044	0.0044	0.0049	0.6166	0.6351	0.6361
	1.71	10714	98.7	0.0046	0.0047	0.0051	0.6095	0.6426	0.6355
	1.96	7213	99.0	0.0056	0.0056	0.0077	0.6209	0.6509	0.7178
	2.29	4590	99.1	0.0062	0.0062	0.0066	0.6410	0.6469	0.6645
	2.44	3806	99.1	0.0073	0.0073	0.0088	0.6586	0.6817	0.7313
	2.67	2936	98.9	0.0067	0.0066	0.0084	0.6474	0.6794	0.7364
	2.81	2528	98.7	0.0074	0.0071	0.0079	0.6671	0.6803	0.6991
	2.92	2261	98.6	0.0069	0.0069	0.0077	0.7049	0.7231	0.7501
	3.64	1180	97.4	0.0072	0.0076	0.0088	0.7269	0.7214	0.7961
	3.76	1081	97.4	0.0081	0.0080	0.0083	0.7926	0.7655	0.7644
	3.98	913	97.3	0.0083	0.0082	0.0096	0.8459	0.8548	0.8597

PDB ID (ref)	Synth. Res. (Å)	No. reflect.	completeness	RMS bond (Å)			RMS angle (deg.)		
				0	0+e	0+H	0	0+e	0+H
3EZM (S22)	1.74	11074	98.4	0.0044	0.0050	0.0050	0.7084	0.7379	0.7371
	1.85	9262	98.8	0.0053	0.0057	0.0057	0.7322	0.7680	0.7530
	2.01	7272	99.0	0.0058	0.0062	0.0065	0.7528	0.7855	0.7841
	2.19	5660	99.2	0.0062	0.0063	0.0065	0.7313	0.7580	0.7506
	2.39	4382	99.3	0.0064	0.0067	0.0073	0.7537	0.7786	0.7945
	2.58	3502	99.4	0.0067	0.0071	0.0069	0.7394	0.7642	0.7548
	2.69	3095	99.4	0.0067	0.0069	0.0083	0.7502	0.7709	0.8539
	2.83	2652	99.5	0.0065	0.0072	0.0076	0.7153	0.7687	0.8170
	2.96	2326	99.4	0.0073	0.0076	0.0078	0.7396	0.7594	0.7586
	3.07	2092	99.4	0.0070	0.0076	0.0077	0.7398	0.7645	0.8211
	3.36	1596	99.4	0.0067	0.0071	0.0067	0.7209	0.7539	0.7663
	3.65	1237	99.4	0.0068	0.0053	0.0080	0.6714	0.6356	0.7814
	3.92	993	99.2	0.0069	0.0067	0.0080	0.7129	0.7439	0.7781
1TP6 (S23)	1.66	12010	99.5	0.0046	0.0050	0.0054	0.6452	0.6806	0.6797
	1.77	9951	99.6	0.0046	0.0050	0.0059	0.6272	0.6601	0.6908
	1.90	8083	99.6	0.0050	0.0052	0.0070	0.6628	0.6860	0.7631
	2.02	6740	99.7	0.0064	0.0065	0.0067	0.6789	0.7066	0.7063
	2.15	5593	99.6	0.0057	0.0056	0.0069	0.6825	0.6971	0.7225
	2.31	4530	99.5	0.0064	0.0065	0.0086	0.6855	0.6946	0.8016
	2.47	3701	99.5	0.0068	0.0070	0.0076	0.6639	0.6931	0.7334
	2.62	3115	99.5	0.0078	0.0072	0.0087	0.7429	0.7162	0.7884
	2.74	2721	99.4	0.0071	0.0069	0.0085	0.6222	0.6456	0.7427
	2.87	2385	99.3	0.0067	0.0070	0.0088	0.6807	0.7028	0.7778
1V8E (S24)	1.64	28505	97.9	0.0049	0.0051	0.0054	0.7446	0.7706	0.7667
	1.81	21489	98.7	0.0053	0.0054	0.0057	0.7543	0.7697	0.7694
	1.99	16357	99.2	0.0058	0.0061	0.0063	0.7634	0.7815	0.7778
	2.20	12196	99.4	0.0063	0.0064	0.0073	0.7813	0.7951	0.8209
	2.36	9926	99.4	0.0067	0.0067	0.0075	0.7836	0.7916	0.8171
	2.52	8181	99.4	0.0065	0.0065	0.0077	0.7782	0.7917	0.8420
	2.66	6975	99.3	0.0068	0.0067	0.0070	0.8015	0.8045	0.8136
	2.82	5880	99.3	0.0067	0.0067	0.0069	0.7809	0.8013	0.7975
	3.01	4858	99.2	0.0071	0.0070	0.0077	0.7762	0.7817	0.8090
	3.13	4329	99.1	0.0070	0.0070	0.0079	0.7664	0.7686	0.8142
	3.46	3234	99.0	0.0077	0.0074	0.0080	0.7712	0.7759	0.8280
	3.78	2495	98.9	0.0079	0.0080	0.0095	0.7572	0.7810	0.8879
	4.11	1942	98.5	0.0071	0.0069	0.0075	0.7191	0.7271	0.7457
	4.39	1607	98.3	0.0087	0.0088	0.0103	0.8309	0.8431	0.9346
1OI7 (S25)	1.49	41792	99.1	0.0044	0.0057	0.0069	0.7336	0.8524	0.9316
	1.71	27656	99.2	0.0052	0.0051	0.0055	0.7411	0.7328	0.7511
	1.85	21836	99.2	0.0051	0.0065	0.0065	0.7191	0.9110	0.8454
	2.02	16758	99.1	0.0078	0.0056	0.0079	0.8973	0.7553	0.8626
	2.18	13321	99.0	0.0078	0.0062	0.0072	0.8603	0.7530	0.8315
	2.37	10357	99.0	0.0090	0.0106	0.0102	0.8943	0.9190	0.9071
	2.54	8397	98.9	0.0076	0.0092	0.0074	0.8999	0.9325	0.7751
	2.69	7086	98.9	0.0093	0.0109	0.0106	0.9054	0.9192	0.8989
	2.84	6014	98.7	0.0074	0.0107	0.0101	0.7799	0.9689	0.9207
	2.97	5256	98.6	0.0077	0.0100	0.0106	0.7734	0.9556	0.9393
	3.28	3897	98.5	0.0086	0.0074	0.0095	0.9176	0.7970	0.9030
	3.57	3003	98.2	0.0100	0.0108	0.0104	0.9481	0.9552	0.9512
	3.83	2425	97.9	0.0099	0.0102	0.0104	0.9296	0.9634	0.9400
	4.07	2015	97.7	0.0067	0.0103	0.0102	0.8860	0.9213	0.9408
